# Anti-Cancer Potential of Cannabinoids, Terpenes, and Flavonoids Present in Cannabis

**DOI:** 10.3390/cancers12071985

**Published:** 2020-07-21

**Authors:** Andrea M. Tomko, Erin G. Whynot, Lee D. Ellis, Denis J. Dupré

**Affiliations:** 1 Department of Pharmacology, Faculty of Medicine, Dalhousie University, Halifax, NS B3H 4R2, Canada; andrea.tomko@dal.ca (A.M.T.); erin.whynot@dal.ca (E.G.W.); 2 Aquatic and Crop Resource Development Research Center, National Research Council of Canada, Halifax, NS B3H 3Z1, Canada; lee.ellis@nrc-cnrc.gc.ca

**Keywords:** cancer, cannabis, cannabinoid, terpene, flavonoid, cytotoxicity, entourage effect

## Abstract

In recent years, and even more since its legalization in several jurisdictions, cannabis and the endocannabinoid system have received an increasing amount of interest related to their potential exploitation in clinical settings. Cannabinoids have been suggested and shown to be effective in the treatment of various conditions. In cancer, the endocannabinoid system is altered in numerous types of tumours and can relate to cancer prognosis and disease outcome. Additionally, cannabinoids display anticancer effects in several models by suppressing the proliferation, migration and/or invasion of cancer cells, as well as tumour angiogenesis. However, the therapeutic use of cannabinoids is currently limited to the treatment of symptoms and pain associated with chemotherapy, while their potential use as cytotoxic drugs in chemotherapy still requires validation in patients. Along with cannabinoids, cannabis contains several other compounds that have also been shown to exert anti-tumorigenic actions. The potential anti-cancer effects of cannabinoids, terpenes and flavonoids, present in cannabis, are explored in this literature review.

## 1. Introduction

Archaeobotanical evidence and written records found in ancient texts of Ayurvedic medicine and the first known Pharmacopoeia “Shen Nung Pen Ts’ao Ching” describe medical use of cannabis for several thousand years. Cannabis use, for religious/spiritual, food, and textile has been documented in written history back to at least the third millennium BC, and potentially even earlier by archaeological evidence (history reviewed in [1,2]). Cannabis reached South America in the mid-1500s and North America in the early 1600s. During the development of Western Medicine, a progressive understanding of cannabis properties led to wider medical and recreational consumption in the 20th century until the use of this plant became marginalized and criminalized, largely due to misinformation, which greatly impacted progress regarding the understanding of the medicinal benefits of this plant and its components [3,4].

The canonical endocannabinoid system is comprised of the main endocannabinoids anandamide and 2-arachidonoylglycerol and cannabinoid receptors CB1 and CB2. Additional components, such as the endocannabinoid-degrading enzymes fatty acid amide hydrolase and monoacylglycerol lipase, other cannabinoid-activated G protein-coupled receptors and members of the transient receptor family, among others, could also contribute to the effects of cannabinoids, and are therefore identified as possible targets involving this class of compounds. The effects of the endocannabinoid system and its potential involvement in cancer have been discussed in several recent publications and only some highlights of the general functions and effects related to cancer are therefore provided here [5,6,7]. Dysregulation of the endocannabinoid system has been implicated in several diseases, including cancer. This dysregulation can include variation in the expression and/or function of cannabinoid receptors and enzymes, or simply alterations the concentration of endocannabinoids [8,9]. For example, dysregulation of cannabinoid receptor levels in malignant tissues has been observed [10] and was associated with poor prognosis for patients with different types of cancer [11,12,13,14,15,16,17]. The levels of endocannabinoids have also been shown to be dysregulated in malignant tissues. For example, concentrations of AEA and 2-AG were increased in colorectal carcinomas when compared with healthy neighboring tissue [18,19,20,21]. Early investigations into the functional implication of endocannabinoids during tumor progression demonstrated that endocannabinoids had inhibitory effects on the proliferation of breast or prostate cancer cells [22,23]. Based on the preliminary evidence in various models, it appears that cannabinoids target key signaling pathways involved in all the hallmarks of cancer [24]. In several indications, cannabinoids complement conventional chemotherapeutic regimens by reducing some of their adverse effects such as pain, nausea, and vomiting. Additionally to the cannabinoids, a large number of terpenes and flavonoids, some of them also present in cannabis, exhibit cytotoxicity against a variety of cancers [25,26]. Fundamental research will allow us to better understand the interrelationship between the various compounds present in the cannabis plant, the endocannabinoid system and cancer. Research will therefore help identify intracellular signaling pathways that participate in cannabinoid anticancer action, and will help discern in which circumstances these compounds should be best tested in clinical trials (reviewed in [27]) for eventual therapeutic use; whether as single therapy agents, synergistically with a validated chemotherapeutic agent or in polypharmaceutical formulations. Below is summarized the current knowledge about the potential effects of cannabinoids, terpenes and flavonoids present in cannabis, as anticancer agents.

## 2. Cannabinoids

**Figure 1:**
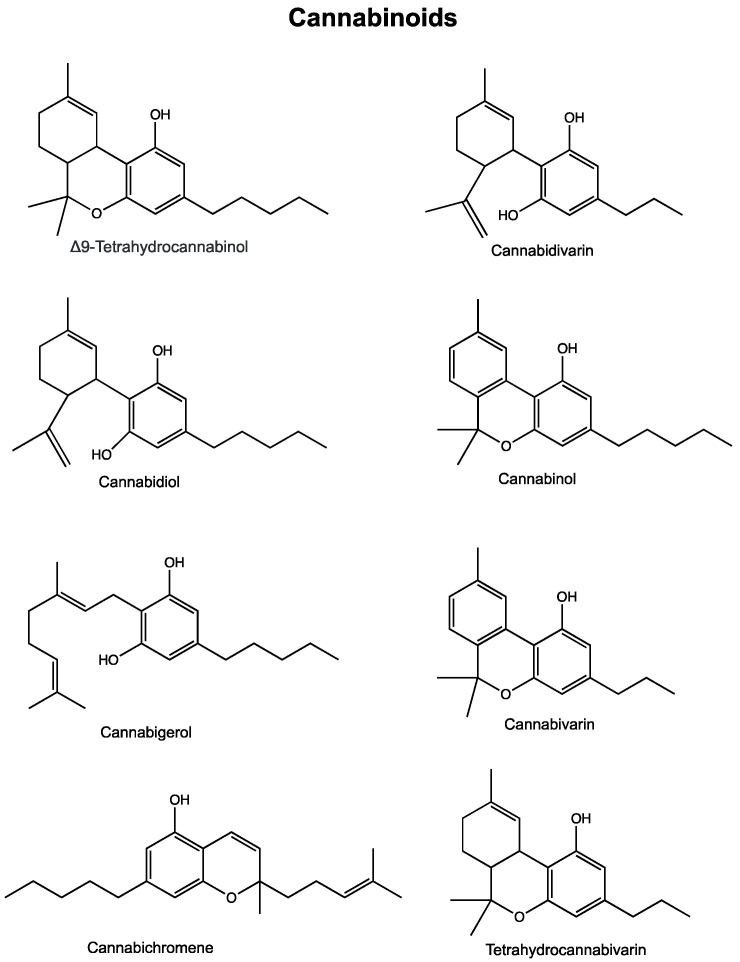
Structure of various cannabinoids found in the Cannabis plant.

More than 100 cannabinoids have been isolated from the plant *Cannabis sativa* [28]. Cannabinoids derive from cannabigerolic acid and differ mainly in the way this precursor is cyclized (Figure 1). Phytocannabinoids can be found in other plant species besides cannabis. These include several types of *Echinacea, Acmella oleracea, Helichrysum umbraculigerum* and *Radula marginata* [29]. Due to its psychoactive effects, the phytocannabinoid tetrahydrocannabinol (THC) is the best-known phytocannabinoid and the primary intoxicating compound in cannabis. Cannabinol also displays intoxicating effects. Most other phytocannabinoids are not intoxicating, the best known being cannabidiol, but also include others, such as cannabigerol, cannabivarin, cannabichromene. The effects of cannabinoids have been examined for various conditions, and we highlight here some of their effects in cancer (Table 1). Considering all the available literature at this time, much stronger experimental evidence (obtained in vitro, in vivo and even in a few clinical trials) support that THC and cannabidiol (CBD) have better anticancer activity than for the other cannabinoids.

### 2.1. Delta9-tetrahydrocannabinol (THC)

Δ^9^-tetrahydrocannabinol (THC) is the major psychoactive component present in *Cannabis sativa* L. cultivars, mediating its effects in the central nervous system via CB1 receptors [126]. THC binds and activates CB1 receptors in the central nervous system (CNS), leading to the intoxicating feelings associated with cannabis use. THC can be administered via multiple routes, including orally, intravenously, intramuscularly and inhalation. The most common method of administration in humans is orally, and due to its high lipophilicity, it is highly bound by plasma proteins and is readily distributed to vascularized tissues such as the liver, heart and lungs. Fat tissues have also been shown to be reservoirs for THC accumulation. Due to the psychoactive effects of THC mediated in the CNS, there are concerns in terms of prescribing THC for medicinal use in cancer patients. There are also other undesirable side effects of THC use, such as dependence, tolerance and issues surrounding abuse [27]. Despite the limitations and concerns associated with THC treatment, there are many studies regarding THC’s potential as an anti-cancer therapy and we highlight these studies herein.

#### 2.1.1. Breast Cancer

In breast cancer cells, THC at a concentration of 14 µM inhibited overall cell growth and proliferation [30]. Exposure to THC was shown to inhibit estradiol-induced cell proliferation by inhibiting estrogen receptor α activation [31]. THC exposure antagonized 17β-estradiol-induced proliferation, and did not act on androgen or estrogen receptors in MCF-7 cells [33]. In contrast, Takeda et al. found that THC increased human epidermal growth factor 2 (HER2) expression, which is able to stimulate cancer cell proliferation, and that THC had proliferative actions in MCF-7 cells [34]. Similarly, a study by McKallip et al. [35] found that treatment of tumors with low levels of cannabinoid receptor expression with THC can actually lead to increased tumour growth and did not induce cytotoxicity in these cells. In addition, they showed that 4T1 mouse mammary carcinoma cells were also resistant to THC, and treatment of these cells in vivo with THC resulted in increased tumor growth and metastasis as a result of suppression of the specific anti-tumor response. Mechanistically, THC’s anti-cancer effects in breast cancer can be mediated by modification of JunD, a transcription factor. THC was shown to activate JunD by both translocating it to the nucleus and up-regulating its expression [32]. This was confirmed by testing THC in breast cancer cells with silenced JunD and JunD knockout mice-derived fibroblasts, where the anti-proliferative effects of THC were significantly reduced. Another study showed that THC reduced human breast cancer cell proliferation via stimulation of CB2 receptors. THC treatment inhibited the cell cycle progression in breast cancer cells at the G2/M phase, which was attributed to the down-regulation of Cdc2, and induced apoptosis [36].

The ability of THC to treat ErbB2-positive breast cancer, a very aggressive form of cancer has been evaluated. In a mouse model of ErbB2-driven metastatic breast cancer, THC treatment was able to reduce tumor growth, as well as the amount and severity of lung metastases. THC treatment also induced apoptosis and limited tumor angiogenesis [40]. Heteromerization of HER2 receptors with CB2 receptors has been shown to control the oncogenic activity of HER2 and is connected to poor patient prognosis [41]. THC treatment disrupted HER2-CB2 receptor heteromers via the binding of CB2, which ultimately resulted in anti-tumor actions both in vitro and in vivo. In a xenograft murine model, THC treatment significantly reduced tumor growth and resulted in decreased expression of the HER2 protein. In MDA-MB-231 breast cancer cells, THC was shown to cause fatty acid 2-hydroxylase (FA2H) induction [37]. A possible mechanism mediating this increase in FA2H is the induction of peroxisome proliferator-activated (PPAR) α [127]. Higher levels of FA2H expression has been reported to be a crucial biomarker that is related to the prognosis of patients with triple-negative breast cancer, where higher levels of FA2H has been shown to result in shorter disease-free survival [37]. Recently, the effects of pure THC versus a botanical drug preparation in the treatment of breast cancer were evaluated. It was found that the whole botanical drug preparation was more potent in terms of anti-tumor action in cellular and murine models of breast cancer. In vivo, THC was found to be less potent at inhibiting tumor growth than the botanical extract. Pure THC produced anti-cancer effects via CB2 and the production of reactive oxygen species, and the botanical preparation exerted its effects through different mechanisms of action [38]. THC has also been shown to significantly inhibit human P-glycoprotein (P-gp) and breast cancer resistance protein (BCRP), which implicates its possible use in reducing resistance to chemotherapeutic agents [39].

#### 2.1.2. Glioma

In glioma cell lines, treatment with THC produced dose-dependent inhibition of cell viability and proliferation [42]. In glioma cells, treatment with THC at 3 µM was able to inhibit cell growth [43]. THC did not have any effects on cell viability in C6 glioma cells when cultured with 10 percent FBS, while it did have modest activity on inhibiting cell viability in a serum-free culture environment [44]. In C6 glioma cells, THC had an IC_50_ of 23 µM and THC exposure increased cell death as a result of oxidative stress [45]. On the contrary, other studies have shown that human glioma cells were only sensitive to THC at very high, pharmacologically irrelevant concentrations, and that it has the potential to stimulate glioma cell growth [50,128]. An early study by Sánchez and colleagues [46] demonstrated THC’s ability to induce apoptosis in human C6 glioma cells and they suggested that this effect may be mediated through a CB1 receptor-independent pathway involving the stimulation of sphingomyelin breakdown. It has been shown that THC induced autophagy-mediated cell death in glioma cells as a result of ceramide accumulation and endoplasmic reticulum stress [51]. Tribbles homolog 3 (TRB3) linked ER stress to autophagy, and autophagy occurred prior to apoptosis in cannabinoid-induced glioma cell death. This sequence of events was required for the in vivo anti-cancer effects of cannabinoids [51,52]. One study found that long-term exposure to THC did not stimulate apoptosis and actually diminished the sensitivity of astrocytes to ceramide accumulation [129]. Carracedo and colleagues [47] showed that stress protein p8 up-regulation and endoplasmic reticulum stress were necessary for THC-induced apoptosis in glioma cells and cells from human astrocytoma biopsies. In cannabinoid-resistant tumor cells, p8 upregulation did not occur following cannabinoid treatment. They also showed that THC treatment upregulated p8 levels in tumors in vivo, and that tumors deficient in p8 were resistant to the apoptotic effects of cannabinoids. Another study showed that THC treatment modified sphingolipid ratios in the endoplasmic reticulum of glioma cells, which resulted in the promotion of autophagy-dependent lysosomal membrane permeabilization and cathepsin release, resulting in the activation of the mitochondrial apoptotic pathway [48]. Administration of THC also reduced glioma tumor growth in vivo as a result of autophagy-mediated cell death.

THC treatment was able to regress malignant glioma tumors in vivo in murine models. Mice with C6 glioma xenografts were treated for 7 days with THC, which was able to increase survival and reduce tumor progression. THC stimulated apoptosis in glioma cells by accumulation of ceramide and Raf1/extracellular signal-regulated kinase activation [49]. Temozolomide (TMZ) combined with THC synergistically reduced the growth of glioblastoma multiforme xenografts when administered locally [54,55,56]. The combination of TMZ with both THC and CBD together was also looked at, and cannabinoid combinations with higher CBD had a stronger anti-tumor effect in xenografts derived from GBM patients. The same group looked at systemic administration of Sativex-like extracts (1:1 THC:CBD) in combination with TMZ, and anti-tumor effects glioma cell-derived tumor xenografts were still observed. THC resistance can occur in glioma cells, and growth factor midkine (Mdk) has been shown to be involved in this resistance. In vivo, silencing Mdk was able to sensitize resistant tumors to the anti-cancer effects of THC, indicating Mdk as a potential target for improving the effectiveness of cannabinoids in glioma treatment [56]. The local administration of THC downregulated the expression of metalloproteinase-2 (MMP-2) in mice bearing glioma tumors. Likewise, in cultured glioma cells, THC exposure inhibited the expression of MMP-2 and reduced invasion, indicating that reduction in MMP-2 plays a fundamental part in THC-induced reduction of cell invasion. Tissue inhibitors of metalloproteinases, for example MMP-1 (TIMP-1), have been shown to be down-regulated by THC exposure, both in vitro and in vivo, which may explain the THC-induced inhibition of metalloproteinases in glioma cells [53]. THC as a botanical drug substance was more effective than pure THC alone in a murine glioma model [106]. Pre-treatment of glioma cells with pure THC and CBD together increased the sensitivity of glioma cells to radiation therapy both in vitro and in vivo due to increased apoptosis and autophagy [106]. Similarly, another study suggested that adding CBD to the THC treatment of glioblastoma cells may improve the overall efficacy of THC in glioblastoma therapy, as the combination of both cannabinoids had synergistic anti-cancer effects [43]. THC-loaded microparticles for the systemic delivery of cannabinoids have also been developed, and this method of THC delivery facilitated prolonged release for several days, and in mice bearing glioma xenografts the microparticles limited cell proliferation and angiogenesis and increased apoptosis [57].

#### 2.1.3. Leukemia

In a leukemia model, THC had an IC_50_ of 13 µM and CBD had an IC_50_ of 8 µM [62]. When THC and CBD were combined in a 1:1 ratio, the IC_50_ was decreased to 4 µM. They then combined THC/CBD combinations with other anti-cancer agents and in some cases observed synergistic effects, but most importantly, they observed that equivalent anti-cancer effects can still result from lower concentrations of combined agents, compared to each agent alone at a higher concentration. Combinations of THC and CBD also slightly sensitized leukemic cells to anti-cancer agents, vincristine and cytarabine. The beneficial effects of combined therapy with cannabinoids and chemotherapeutic agents was dependent on the sequence of administration; increased cell death was observed when cannabinoids were administered after chemotherapy [62]. THC sensitized leukemia cells to well-established anti-cancer agents, decreasing the IC_50_ values by approximately 50 percent. The sensitization was found to be due to THC’s ability to down regulate phosphorylated ERK [63]. This data supports the notion that cannabinoids combined with other therapeutic agents may enhance the overall anti-cancer effects. Inhibition of the differentiation blockage in acute myeloid leukemia has been shown to be the most successful target in leukemia therapy. Dronabinol, the enantiomer (−)-*trans*-Δ^9^-tetrahydrocannabinol approved by the FDA for conditions like HIV/AIDS-induced anorexia and chemotherapy-induced nausea and vomiting, was found to inhibit the differentiation blockage in acute leukemia cells in vitro and that O-linked-β-*N*-acetyl glucosamine transferase was fundamental to this process [65].

Dronabinol also reduced cell viability and proliferation, as well as induced apoptosis in an array of acute leukemia cell lines and native leukemic cells cultured ex vivo. Dronabinol’s pro-apoptotic effects in patient-derived leukemic cells correlated with expression of CB1 and CB2 receptors, where the presence of these receptors was necessary to see apoptotic effects. The response to THC treatment was found to be higher in leukemia blasts that were derived from a lymphatic lineage and expressed lymphatic markers [66]. One study suggested that THC-induced apoptosis in leukemia cells occurred as the result of BCL2 associated agonist of cell death (BAD) translocation to the mitochondria. Use of a BAD siRNA was able to reduce THC-induced apoptosis in leukemia cells [67]. Another study used Jurkat leukemia cells with defects in signaling pathways to determine the mechanism of apoptosis induced by THC exposure. The intrinsic pathway was found to play a fundamental role in THC’s ability to induce apoptosis in Jurkat cells [68]. THC also decreased P-gp expression in CEM/VLB(100) cells, which correlated with an increase in the accumulation of P-gp substrate Rh123 and sensitized the cells to vinblastine [64].

#### 2.1.4. Lung Cancer

Low levels of THC induced lung cancer cell proliferation. Metalloprotease and epidermal growth factor receptor (EGFR) activity were found to be fundamental in mediating this increase in cell proliferation [50]. Another study looked at the anti-tumor effects of whole cannabis extracts versus individual compounds alone. In lung cancer cells, they found that treatment with pure THC did not significantly decrease cell survival, relative to control [70]. In contrast, other studies found that THC inhibited epidermal growth factor (EGF) stimulated growth of non-small cell lung cancer and reduced the expression of EGFR, as well as chemotaxis and invasion [71,72]. THC inhibited contact-dependent macrophage cell killing of tumor cells in a cannabinoid-receptor independent manner [73]. Similarly, THC treatment suppressed host immune reactivity to lung cancer and in murine models of lung cancer, administration of THC caused increased tumor growth and decreased tumor immunogenicity [75]. A different study found that THC was able to inhibit tumor growth and lung metastases in a murine model of lung cancer [71]. In non-small cell lung cancer (NSCLC) cells, treatment with THC was able to suppress the epithelial-mesenchymal transition, restore the epithelial phenotype and reduced the proliferation of these cells in vitro. In addition, THC reduced the migration of NSCLC cells [72]. THC-loaded nanoparticles for the treatment of lung cancer caused significant cytotoxicity against human and murine lung cancer cells in vitro and in vivo [74].

#### 2.1.5. Melanoma

Human melanomas have been shown to express both CB1 and CB2 receptors. In melanoma cells, stimulation of these receptors by THC decreased cell viability, proliferation, metastasis, angiogenesis and induced the activation of autophagy and apoptosis [76,78,80]. Treatment with THC was able to diminish the survival of mitogen-activated protein kinase inhibitor (MEKi)-resistant melanoma cells. The combined treatment with a MEK inhibitor (Trametinib) and THC reduced cell viability, invasion and metastasis of MEKi-resistant melanoma cells in vivo [80]. THC has been shown to reduce melanoma cell proliferation and tumor growth in vivo in murine models in other studies [76,79]. In one study, a preparation of equal amounts THC:CBD was able to decrease tumor growth and increase autophagy and apoptosis in vivo [78]. THC significantly inhibited the tumor growth of transplanted mouse melanoma cells in a cannabinoid receptor-dependent fashion [79].

#### 2.1.6. Myeloma

THC has been demonstrated to exert anti-cancer effects in multiple myeloma (MM) cells. THC at concentrations ranging between 30–40 µM was able to inhibit cell viability and proliferation and induce autophagic-dependent necrosis of MM cells. One study found that the combination of THC and CBD had the most potent effects in MM cells compared to each compound on its own. THC and CBD together had synergistic effects with carfilzomib, increasing cell death and decreasing migration. THC treatment alone also reduced migration of MM cells via decreasing the expression of CXCR4 and CD147 (plasma membrane glycoprotein) [77]. Myeloid-derived suppressor cells (MDSC) are induced by cancers with the purpose to evade anti-tumor immune responses and have been shown to be increased in patients with multiple myeloma [81,130]. THC induced MDSCs in mice via epigenetic alterations that promoted MDSC function and differentiation, and S100A8 (a calcium-binding protein) was shown to play a key role in this process [81].

#### 2.1.7. Hepatocellular Carcinoma

THC treatment reduced the viability of hepatocellular carcinoma (HCC) cells in vitro in a CB2-dependent manner. THC also induced autophagy in HCC cells, which was found to be reliant on tribbles homolog 3 (TRB3) up-regulation, and was able to reduce tumor growth in a xenograft murine model [82]. Another study also showed that TRB3 plays a fundamental role in the anti-cancer action of THC, where transformed embryonic fibroblasts derived from TRB3-deficient mice were resistant to the effects of THC [131]. THC increased the activity of PPARγ in HCC cells, and the pharmacological inhibition of PPARγ inhibited the anti-tumor action of THC in these cells, both in vitro and in vivo, indicating that the anti-proliferative actions of THC in HCC cells are influenced by PPARγ-dependent pathways [83]. In a Wistar rat model, co-treatment of irinotecan with THC led to decreased hepatic toxicity, which suggested a protective role of THC on liver injury during acute treatment [85]. In cholangiocarcinoma cells (including a patient sample) THC exposure suppressed proliferation, migration and invasion, and induced apoptosis [84].

#### 2.1.8. Pancreatic Cancer

In pancreatic cancers, cannabinoid receptors have been shown to be much more highly expressed than in regular tissues. Treatment of pancreatic cancer cells with THC was able to decrease cell viability, induce apoptosis, and reduce the growth of tumors in vivo in murine models. The induction of apoptosis by THC in pancreatic cancer cells was the result of ceramide accumulation and endoplasmic reticulum stress, as shown by increased expression of stress protein p8 mRNA [86].

#### 2.1.9. Prostate Cancer

In human prostate cancer cells, treatment with THC was able to reduce cell viability [87]. The IC_50_ of THC in DU-145 prostate cancer cells was greater than the highest concentration tested (25 µM) [30]. A study found that THC induced apoptosis in a dose-dependent and cannabinoid receptor-independent manner in prostate cancer cells [88].

#### 2.1.10. Colon Cancer

In colorectal cancer cells, THC had an IC_50_ of 17 µM [30]. Another study found that THC induced apoptosis in colorectal cancer cells via the activation of CB1 receptors and subsequent inhibition of PI3K-AKT and RAS-MAPK/ERK survival pathways [89]. THC biodegradable microspheres were developed for an alternative cannabinoid delivery system than the usual oral route. The THC microspheres were able to inhibit cell proliferation of multiple cancers, including Caco-2 colon cancer cells, over a 9 day period [90].

#### 2.1.11. Endometrial and Cervical Cancers

There are currently very limited options for treatment of aggressive endometrial cancer, and it has been shown that cannabinoid receptors are highly expressed in endometrial cancer tissues. THC treatment of endometrial cancer cells decreased cell viability and motility as a result of inhibiting the epithelial-mesenchymal transition and decreasing metalloproteinase-9 gene expression [132]. ATP-binding cassette (ABC) transporters are highly implicated in the resistance of cancers to anti-cancer drugs. THC was able to increase the accumulation of Fluo3 and Vincristine in ovarian cancer cells that over-expressed ABCC1, both of which are substrates for the transporter [91]. In another study, a different multidrug transporter, ABCG2, was shown to be inhibited by THC, where THC exposure increased the accumulation of mitoxantrone, an ABCG2 substrate [92]. In cervical cancer cells, one study found that THC decreased invasion via the increased expression of tissue inhibitor of MMP-1 (TIMP-1) [93]. This suppression of invasion was reversed by the knockdown of THC-induced TIMP-1 expression.

#### 2.1.12. Oral Cancer

In human oral cancers that are highly resistant to anti-cancer drugs, exposure to THC significantly inhibited mitochondrial oxygen consumption and exhibited strong toxicity in these highly malignant cells [94].

#### 2.1.13. Clinical Results

Inglet et al. [133] recently comprehensively outlined clinical data supporting the use of cannabis-based treatments in a variety of disease states, including cancer. Afrin et al. [27] also presented completed, ongoing, and recruiting clinical trials looking at the effects of cannabis-based treatment in cancer patients. Completed clinical trials that incorporated THC as treatment for various cancers are presented below. One study found that in two patients with pilocytic astrocytomas, tumors regressed over a period of 3 years and neither patient was receiving any conventional adjuvant treatment; however, cannabis was consumed via inhalation over the same period, suggesting cannabis played a role in tumor regression. Unfortunately, no details were available regarding the type, strength, or frequency of cannabis use in these patients [58]. In another study, a patient with terminal acute lymphoblastic leukemia was given *Cannabis sativa* oil (normally higher in THC content than other cannabinoids), and was able to achieve remission, attributed to the effects of the cannabis oil as the patient was solely on cannabinoid treatment [69]. A two-part clinical study in 2016 investigated the effects of TMZ and Sativex (THC:CBD 1:1) in glioblastoma multiforme patients and found that combination treatment was able to increase the 1 year survival rate by 39 percent (NCT01812603 and NCT01812616). A pilot clinical study looked at the potential of THC treatment in patients with recurrent glioblastoma multiforme. THC inhibited tumor cell proliferation in vitro and reduced tumor cell Ki67 immunostaining when given to 2 patients [59]. In another study, intratumoral injection of THC in two patients suffering from glioblastoma multiforme was able to decrease vascular endothelial growth factor (VEGF) levels, as well as decrease VEGF-2 receptor activation [60]. Dronabinol has had limited use in central nervous system cancers due to the risk of CNS adverse events, however a recent study reported that participants with primary brain tumours did not experience adverse events to the same severity that other studies have reported. This study was however limited by the low number of participants, and the dronabinol dosage used was low (10 mg) relative to other studies [61]. Between 2000 and 2010, pediatric cancer patients receiving chemotherapy were also given low doses of Dronabinol to determine its potential use as an adjuvant antiemetic in children. They found that 60 percent of patients had a positive response to Dronabinol [134]. Dronabinol also inhibited the differentiation blockage in two patients suffering from leukemia [66]. Finally, a study tested the effects of THC in cancer patients in palliative care, and found that daily doses were generally well-tolerated, and nearly 50 percent of patients experienced overall improvement in their well-being [135].

### 2.2. Cannabidiol (CBD)

Cannabidiol (CBD) is one of the major and most extensively researched phytocannabinoids present in cannabis species. Cannabidiol binds to a large array of physiological targets within the body’s endocannabinoid system. In medical settings, CBD is most commonly administered orally and is commonly prepared as an oil. Cannabidiol is non-intoxicating, which is why its potential as a therapeutic agent is more appealing than some other cannabinoids that do possess psychoactive effects, like THC. To date, there are many studies surrounding the anti-cancer potential of cannabidiol. Two recent reviews by Afrin et al. and Kis et al. [27,136] thoroughly highlighted research studies investigating the anti-cancer effects of cannabidiol. Treatment with CBD exhibited a multitude of beneficial anti-cancer effects in lung, breast, colon, prostate, melanoma, leukemia, cervical, brain, neuroblastoma and multiple myeloma cancer cells (Reviewed in [27,136]), and we highlight these studies here.

#### 2.2.1. Breast Cancer

Several studies have examined the effects of CBD in vitro and in vivo in breast cancer. In breast cancer cells, treatment with CBD has been shown to induce apoptosis and autophagy [30,95]. It has been suggested that CBD can induce endoplasmic reticulum stress apoptosis by enhancing the production of reactive oxygen species (ROS) in select breast cancer cells [95]. Another study demonstrated CBD’s ability to inhibit epidermal growth factor (EGF)-induced proliferation, migration and invasion of breast cancer cells [97]. Recently, CBD’s ability to inhibit the epithelial-mesenchymal transition (EMT) in cancer cells has been an emerging area of research. CBD can revert the EMT in highly invasive breast cancer cells. Treatment of 6D breast cancer cells with CBD was able to significantly reduce migration and invasion, promoted the recovery of cell contacts, and reduced the expression of malignant markers. CBD was also able to increase sensitivity to anticancer agents doxorubicin and cisplatin in 6D cells by down-regulating the expression of resistance proteins [98]. In murine models of breast cancer, CBD reduced cell proliferation and overall tumour growth, as well as migration and invasion to reduce metastasis [97]. One study reported that CBD treatment reduced advanced-stage breast cancer metastasis via the downregulation of Inhibitor of DNA binding protein 1 (Id1), a transcriptional factor [100]. Another study also showed that CBD treatment was able to reduce proliferation and invasion of breast cancer cells via reducing the expression of Id-1. In breast cancer, Id-1 overexpression has been found to be highly correlated with the ability of primary human breast cancer cells to metastasize to the lung [96]. An interesting study by Fraguas-Sanchez et al. [99] looked at the combination of CBD solution or CBD encapsulated in polymeric nanoparticles with chemotherapeutic agents paclitaxel or doxorubicin in breast cancer cells. They found that co-administration of CBD solution or CBD nanoparticles with paclitaxel or doxorubicin had synergistic effects on antiproliferative activity. CBD nanoparticles were also effective as a monotherapy and had prolonged antiproliferative activity, lasting for 10 days, indicating that they may be beneficial for extended release of the cannabinoid during treatment [99].

#### 2.2.2. Lung Cancer

In lung cancer, CBD has been shown to induce apoptosis via cyclooxygenase 2 (COX2) and PPARγ [101]. Several studies using lung cancer cells demonstrated that CBD inhibited invasion and metastasis via decreased secretion of plasminogen activator inhibitor-1 [102,104]. CBD also upregulated the expression of surface protein intercellular adhesion molecule (ICAM-1) in lung cancer cells, which correlated with decreased metastasis of these cells. Additionally, CBD treatment increased the susceptibility of lung cancer cells to adhere to and subsequently be lysed by lymphokine-activated killer (LAK) cells, and that the upregulation of ICAM-1 was responsible for the increased action of LAK cells [105]. One study examined the effects of CBD on the proliferation, migration and EMT in lung cancer cell lines. They found that CBD treatment restored the epithelial phenotype and reduced migration of lung cancer cells [72]. In vivo mouse models of lung cancer showed that treatment with 10 mg/kg/day of CBD resulted in reduced cell viability, decreased overall tumour growth and decreased metastasis [101,103].

#### 2.2.3. Glioma and Neuroblastoma

In gliomas, cannabidiol has been shown to exert anti-cancer effects. In glioma cells, treatment with CBD inhibited cell proliferation and induced apoptosis [43,107,108,109,110]. An interesting study showed that CBD exhibited dose-dependent reduction of cell viability in glioma cells, and that pure CBD was more effective than CBD as a botanical drug substance [106]. A study by Singer et al. [110] showed that CBD-induced apoptosis in glioma stem cells was the result of increased ROS production. An interesting study found that treatment with CBD increased the expression and abundance of heat shock proteins (HSP) in glioma cells as a result of CBD-induced ROS production. Increases in HSP diminished the cytotoxic effects of CBD; when glioma cells were cultured with CBD and HSP inhibitors, the cytotoxic effects were restored. In addition, culturing glioma cells with CBD and HSP inhibitors increased the radio sensitivity of the cells, compared to treatment with CBD alone [112]. In vivo murine models of brain cancer revealed that treatment with CBD was able to inhibit tumor growth, enhance apoptosis and significantly prolong mouse survival [57,108,110]. López-Valero and colleagues [54,55] looked at the combination of Temozolomide (TMZ) with both CBD and THC together in the treatment of glioblastoma multiforme (GBM) in vivo. They found that treatment of glioma cell derived xenografts with TMZ in combination with THC and CBD in a 1:1 ratio and preparations higher in CBD, but not TMZ with CBD alone, exhibited similar anti-tumor effects. On the contrary, in xenografts derived from glioma initiating cells, the combination of TMZ with cannabinoid preparations higher in CBD had stronger anti-tumor effects [54]. The same group looked at systemic administration of Sativex-like extracts (1:1 CBD:THC) in combination with TMZ, and found that treatment was still able to produce anti-tumor effects glioma cell-derived tumor xenografts [55]. In neuroblastoma cell lines, CBD decreased cell growth, induced cell cycle arrest, reduced invasion, and reduced tumor growth in vivo [111]. Another study showed that CBD induced apoptosis in neuroblastoma cells via serotonin and vanilloid receptor activation, and reduced cell migration and invasion in vitro [109]. CBD-loaded microparticles were also used for the treatment of mice bearing xenograft gliomas, where they decreased cell proliferation and angiogenesis of tumors [57].

#### 2.2.4. Myeloma

In multiple myeloma cells, CBD reduced cell viability, increased cytotoxicity of bortezomib and carfilzomib, and inhibited cancer cell migration [77,119].

#### 2.2.5. Colon Cancer

Cannabidiol reduced cell viability, elevated ROS levels and promoted apoptosis in colon cancer cells [113,123]. CBD significantly reduced the number of aberrant crypt foci polyps and tumors in a mouse model of colon cancer. CBD had a chemo-preventative effect on colon cancer cells that was the result of up-regulated caspase-3 [113]. Other in vivo studies demonstrated that treatment with CBD reduced colon cancer cell proliferation, induced apoptosis, and also had anti-metastatic and anti-angiogenesis effects [114]. In HCT116 colon cancer cells, CBD’s antagonistic activity at GPR55 was shown to play a key role in the reduction and prevention of metastasis [117]. In vivo models of colorectal cancer found that CBD treatment induced apoptosis by altering the expression of pro- and anti-apoptotic proteins and decreased overall tumor volume [118].

#### 2.2.6. Prostate Cancer

CBD also exhibits multiple promising anti-cancer effects in prostate cancer studies. Treatment with CBD was able to significantly reduce the growth of various prostate cancer cell lines [87,115]. It was reported by De Petrocellis et al. [87] that CBD inhibited growth of prostate cancer cells via the induction of intrinsic pathways of apoptosis, cell cycle arrest at the G1-S phase and activation p53 and elevated ROS levels. CBD treatment was also able to inhibit tumour growth and increase the effects of bicalutamide and docetaxel in a murine xenograft model [87].

#### 2.2.7. Other Cancers

Cannabidiol has been explored for its potential beneficial effects in melanoma, leukemia, cervical, ovarian and endometrial cancers. In mice injected with melanoma cells, treatment with CBD exhibited very similar effects to treatment with anticancer agent cisplatin, such as increasing survival, significantly reducing melanoma tumour growth, and improving overall quality of life [78,116]. A study by Kalenderglou et al. [137] showed CBD’s ability to decrease cell viability and increase the number of cells in the G1 phase in T acute lymphoblastic leukemia cells. Apoptosis was also induced by CBD exposure in leukemic cells as a result of ceramide accumulation [120]. One study found that CBD decreased P-gp expression in CEM/VLB(100) cells, which correlated with an increase in the accumulation of P-gp substrate Rh123, and sensitized the cells to vinblastine [64]. In cervical cancer cells, treatment with CBD ranging from 1.5 µg/mL to 3.2 µg/mL resulted in the inhibition of cell growth and apoptosis [122]. ABC transporters are highly implicated in the resistance of cancers to anti-cancer drugs. In ovarian cancer cells over-expressing ABCC1, CBD exposure was able to increase the intracellular accumulation of 2 ABCC1 substrates, Fluo3 and Vincristine [91]. Another study showed that CBD inhibited multidrug transporter ABCG2 and promoted the intracellular accumulation of mitoxantrone, a substrate for this transporter [92]. In endometrial cancer, concentrations of CBD higher than 5 µM significantly reduced cell viability. CBD increased levels of caspase 3/7, reactive oxygen species and cleaved poly (ADP-ribose) polymerase (PARP) in Ishikawa cells, indicating induction of apoptosis. The activation of transient receptor potential cation channel subfamily V member 1 (TRPV1) was fundamental in facilitating CBD’s anti-cancer effects in endometrial cancer cells [121].

#### 2.2.8. Clinical Results

There have been a few clinical trials involving multiple cancer types that looked at the therapeutic potential of CBD treatment in cancer patients. Afrin et al. [27] highlighted completed, ongoing and recruiting clinical trials looking at effects of cannabinoids in cancer, some of which included CBD treatment. The completed trials that included CBD as treatment are outlined here. In 2016, a two-part clinical trial (NCT01812603 and NCT01812616) was performed to assess the effectiveness of Sativex (1:1 THC:CBD) in combination with Temozolomide in glioblastoma multiforme patients, and found that it was able to increase 1 year survival rate by 39 percent. Another study tested the effects of CBD in cancer patients undergoing palliative care, and found that daily doses of CBD were generally well-tolerated, and 50 percent of patients experienced overall improvement in their condition [135]. They did, however, mention that these results need to be replicated in a trial with placebo controls.

### 2.3. Cannabigerol (CBG)

Cannabigerol (CBG) is one of the main active phytocannabinoids produced by *Cannabis Sativa* L. plants; however, it is considered a minor phytocannabinoid due to its lower abundance relative to THC and CBD. Cannabis cultivars that tend to have higher cannabigerol content are referred to as Type IV cannabis [138]. Cannabigerol is derived from its acidic precursor cannabigerolic acid (CBGA), which also serves as the precursor molecule for the production of THC and CBD. Recently, cannabigerol has attracted more attention for its use in therapeutics due to its lack of intoxicating effects, and more commercial hemp varieties have been developed with CBG and CBGA as the main phytocannabinoids present [139]. As of yet, only a handful of studies have been done to investigate the anti-cancer potential of cannabigerol. Two early studies by Baek et al. [124,125] looked at the potential therapeutic benefits of cannabigerol in mouse skin melanoma cells and oral epithelioid carcinoma (KB) cells. In mouse skin melanoma cells, CBG was found to have significant inhibitory effects on proliferation, with an IC_50_ of 3 µg/mL. In KB cell lines, CBG over a concentration range of 1–100 µM was the most effective of the cannabinoids tested at reducing cell viability. In MDA-MB-231 breast carcinoma cells, 25 µM CBG was shown to inhibit the uptake of [^14^C]anandamide and activate vanilloid receptor TRPV1 [30]. CBG also stimulated apoptosis, ROS production, up-regulated C/EBP homologous protein (CHOP) mRNA and inhibited cell proliferation in colorectal cancer (CRC) cells. In vivo, CBG was shown to decrease the growth of xenograft tumours in a murine model, and that this effect was largely due to its activity as an antagonist at TRPM8 receptors on CRC cells [123].

### 2.4. Cannabichromene (CBC)

Cannabichromene is considered another one of the minor phytocannabinoids produced by *Cannabis sativa* L. plants, due to its lower abundance than the major cannabinoids THC and CBD. In the United States, cannabichromene has been found to be the second most abundant type of cannabinoid present in some strains of cannabis, particularly abundant in dry-type cannabis material. Though CBC is commonly found in many cannabis strains, there is much to be discovered about its pharmacology. Like CBD, cannabichromene lacks intoxicating effects and is therefore appealing to researchers in terms of its potential therapeutic effects in human health and medicine [140]. The potential anti-cancer effects of cannabichromene have not been extensively studied. In prostate carcinoma (PCC) cells DU-145 and LNCaP, cannabichromene was found to be the second most potent inhibitor of cell viability behind CBD, and CBC at 10 µM had very little effect on caspase 3/7 activity. In serum deprived PCC 22RV1 and PC-3 cells, treatment with CBC at 20 µM resulted in significant activation of caspase 3/7, and CBC caused elevated intracellular Ca^2+^ in all four PCC cell lines mentioned [87]. In colorectal cancer Caco-2 cells, CBC was able to inhibit cell growth, but only at a concentration of 30 µM [123]. In MDA-MB-231 and MCF-7 breast cancer cell lines, CBC demonstrated high potency as an inhibitor of cell viability [30].

### 2.5. Cannabidivarin (CBDV)

Cannabidivarin’s structure is similar to cannabidiol except with a shortened side chain. Cannabis cultivars with relatively high levels of CBDV have been identified in India and Nepal. In one study, CBDV was assessed for potential cytotoxic effects on various human prostate carcinoma cell lines. Results showed an IC_50_ of around 20 µM [87]. In colon cancer cells, CBDV inhibited cell viability in a concentration-dependent manner, with an IC_50_ of 10 µM [123].

### 2.6. Cannabinol (CBN)

Cannabinol (CBN) is present in the cannabis plant and, particularly in aged cannabis, is the degraded product of tetrahydrocannabinolic acid. While it was the first of the phytocannabinoids to be isolated, it remains poorly studied. Cannabinol’s psychoactive effects are estimated to be 10 times lower than that of THC [141]. Some cytotoxic effects were observed for cannabinol in prostate cancer cell lines DU-145 and LNCaP. The observed IC_50_ was reported to be superior to the highest dose tested (25 µM) in most experiments [87]. CBN has also been shown to have some antiproliferative effects in aggressive breast cancer cells [96]. Multi-drug transporters are an ongoing issue in the treatment of cancers due to their ability to confer resistance to multiple anti-cancer agents. In one study, CBN inhibited multidrug transporter ABCG2 and promoted the accumulation of mitoxantrone, a substrate for this transporter [92].

### 2.7. Cannabivarin (CBV)

Cannabivarin (CBV), also known as cannabivarol, is found in minor amounts in some cannabis cultivars, and it is an analog of cannabinol with a shortened side chain. It is considered an oxidization product of tetrahydrocannabivarin and is rarely found in fresh cannabis. There does not appear to be any published literature surrounding the biological effects of cannabivarin (or cannabivarol) in cancer.

### 2.8. Tetrahydrocannabivarin (THCV)

Tetrahydrocannabivarin (THCV) is a homologue of THC, where different side chains contribute to a variety of effects that are distinct from THC. Most cannabis cultivars only contain trace amounts of THCV, but some *sativa* cultivars from hybridized African genetics may have higher levels of THCV. As for most other minor cannabinoids, little has been demonstrated regarding the effects of THCV in cancer. Some cytotoxic effects were observed for tetrahydrocannabivarin in prostate cancer cell lines DU-145 and LNCaP, with IC_50_ values above 17.5 µM [87].

## 3. Terpenes

**Figure 2:**
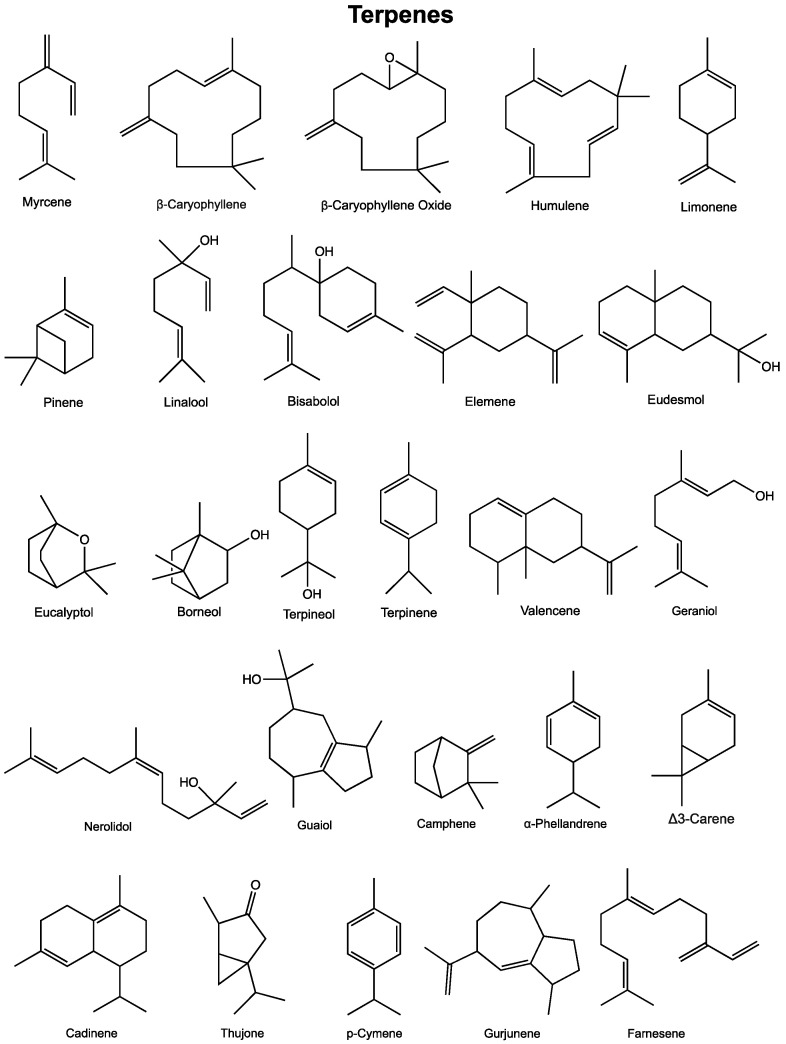
Structure of various terpenes found in the Cannabis plant.

More than 20,000 terpenes appear in nature, from every plant, flower, and even some insects. Relatively few of these compounds–about 200–are found in cannabis. According to recent publications [142,143], 50 cannabis terpenes can be found in North American chemovars, but some are more commonly found (Figure 2). The monoterpene myrcene as well as the sesquiterpenes β-caryophyllene and α-humulene appear to be present in most cannabis cultivars. Other compounds commonly found include alpha-pinene, limonene, linalool, bisabolol and (*E*)-β-farnesene while some others, in particular sesquiterpenes, are difficult to identify. As a result, the reported terpene profiles of cannabis cultivars may present incomplete portraits of the actual terpenes present in the plant [144]. Furthermore, even within a plant, the localization of the sample taken may also alter the terpene profile. Stereochemistry is also not consistently described in cannabis cultivars. These issues make it difficult to fully understand the diversity of terpenes in cannabis and complicates the analysis of studies using extracts or botanical preparations [143]. Generally, terpenes are typically found in cannabis flowers at levels of 2–5%, but can have much higher concentrations in various products (vaping oils, for example). Yet, information about many of the terpenes is available in regard to their potential beneficial effects. Some of those effects, related to cancer, are described below and in Table 2.

### 3.1. Myrcene

Myrcene can be found in food plants, such as hop, bay, verbena, lemongrass, citrus, pomegranate, and carrot. In 2010, the US National Toxicology Program (NTP) published a report on the toxicology and carcinogenesis of β-Myrcene, where it concluded that this chemical can cause cancers in F344/N Rats and B6C3F1 mice (Gavage Studies). Evidence was found for kidney cancers in male rats and liver cancer in male mice [150]. It also appears as though myrcene could produce beneficial effects as extracts from various plants, as these extracts showed significant cytotoxic effects in various tumors including breast carcinoma and colon adenocarcinoma [145], and other cell lines [146]. Cytotoxicity of myrcene against human cervical carcinoma, lung carcinoma, colon adenocarcinoma was also found [147]. In a screen of 12 monoterpenes, myrcene exhibited significant cytotoxicity against leukemia cells [148]. In human B lymphoid NC-NC cells, myrcene reduced t-butyl hydroperoxide induced DNA damage by about 50 percent at 0.01 µg/mL, but was ineffective in human hepatoma [149]. It is highly surprising that so little is known about the potential mechanism of action of myrcene and its potential effects, positive or negative, given the high levels found in cannabis. More work needs to be done with this compound to determine its potential uses or restriction in humans.

### 3.2. Beta-Caryophyllene and Metabolite Caryophyllene Oxide

The sesquiterpene caryophyllene is described to have a spicy or peppery aroma and is found in several plants including black pepper, oregano, cloves, basil and rosemary. Caryophyllene is commonly present in cannabis, and caryophyllene oxide is used for cannabis identification by drug-detecting dogs [305]. Several properties and effects of these compounds in various conditions including cancer have been reviewed by Fidyt et al. and Russo [305,306]. Therefore, only the more recent advances related to the effects of these compounds in cancer are summarized here. β-caryophyllene displayed cytotoxic activity in lung cancer and ovarian cancer cell lines through induction of cell cycle arrest and apoptosis [151,152]. In a glioblastoma model, Irrera et al. [307] demonstrated that the antiproliferative effects of β-Caryophyllene could be blocked by a CB2 receptor antagonist. β-caryophyllene at 20 µM was shown to induce reactive oxygen species, proapoptotic and antiproliferative effects that appeared to be dependent on the activation of the JAK1/STAT3 pathway in osteosarcoma cells [153]. β-caryophyllene and caryophyllene oxide were also tested against multiple other human cancer cell lines. Both substances enhanced the cytotoxicity of doxorubicin, although caryophyllene oxide displayed better results. Similar effects were observed with lymphoblast CCRF/CEM cells and breast cancer cell lines in vitro, but not in vivo. These results suggest a role for these compounds as new chemo-sensitizing agents for doxorubicin chemotherapy and to re-sensitize resistant cancer cells [156,157,158,159]. A possible role of STAT3 as an effector regulated by β-caryophyllene could lead to increased doxorubicin-sensitivity, as shown using cholangiocarcinoma cells [155]. Similar sensitizing effects were found for β-caryophyllene oxide with classical cytostatic drugs, 5-fluorouracil and oxaliplatin in colon cancer cell lines [160] as well as in liver cancer cells treated with sorafenib [161]. Caryophyllenes can also be combined with other compounds from plants to generate effects. In skin epidermoid cancer and precancerous cells, the combination of β-caryophyllene with aromadendrene oxide 2 and phytol, found in *Pamburus missionis*, induced apoptosis, accumulation of cells at the G0/G1 phase. Depending on the combinations, the effects were either additive or synergistic in several skin cancer cell lines [154]. Interestingly, not only did β-Caryophyllene sensitize cancer cells, it was also found to attenuate doxorubicin-induced chronic cardiotoxicity through myocardial CB2 receptor activation in rats [162]. This highlights the therapeutic potential of combination therapy using compounds present in cannabis to reduce some toxic effects of current chemotherapies.

### 3.3. Humulene

Most cannabis varieties display high levels of the monocyclic sesquiterpene humulene. Humulene, formerly known as α-caryophyllene, is described as one of the core cannabis terpenes, along with myrcene, terpinolene, limonene, pinene, and geraniol. Humulene is very common in nature and is responsible for the distinct aroma and flavors of a number of herbs and products. Humulene was first found in the essential oils of *Humulus lupulus* (or common hops), a species of plant in the hemp family that gives beer its distinctive bitter “hoppy” taste. Elsewhere, its aroma is earthy and woody, with spicy, herbal notes. In cannabis, it is found most often in *sativa* strains. A 2003 study [308] showed that in an extract of balsam fir oil, only alpha-humulene had active properties (GI_50_ = 55–73 μM). Balsam fir oil and alpha-humulene caused a dose- and time-dependent increase in ROS production and cytotoxic activity against cell lines including MCF-7, PC-3, A-549, DLD-1, M4BEU and CT-26. Similar effects of alpha-humulene were observed in LNCaP cells with an IC_50_ of 11 μg/mL [163]. Others have found that alpha-humulene was cytotoxic to hepatocellular carcinoma, where it inhibited Akt activation, and therefore decreased GSK-3 and BAD phosphorylation, promoting apoptosis. It also inhibited cell proliferation and increased apoptosis in a hepatocellular carcinoma xenograft model [164]. Interestingly, alpha-humulene can work in concert with other compounds and display synergism. β-caryophyllene was synergistic with humulene in one study; α-humulene or isocaryophyllene alone (32 μg/mL) inhibited cell growth by approximately 50 and 69 percent, respectively. In comparison, when each compound was combined with 10 μg/mL beta-caryophyllene, cell growth was inhibited by 75 and 90 percent, respectively [165]. α-humulene, as well as other terpenes like valencene, β-caryophyllene-oxide and trans-nerolidol, were shown to improve the antiproliferative effect of 5-fluorouracil and oxaliplatin in colon cancer cell lines CaCo-2 and SW-620 [160]. Another study highlighted the potential of humulene in combination with doxorubicin in SKOV3 cells, an ovarian cancer cell line [158].

### 3.4. Limonene

Limonene is a cyclic monoterpene and is the main component of citrus fruit peel oils. Limonene exhibited anticancer effects in several types of cancers in vitro and in vivo by reducing tumor growth and by inducing apoptosis through several mechanisms. Limonene displayed cytotoxic effects in T24 human bladder cancer cells by inducing G2/M phase cell cycle arrest, decreasing migration and invasion, causing apoptosis, increasing Bax and caspase-3 expression and decreasing Bcl-2 expression [166]. In HepG2 cells limonene altered the regulation of genes related to apoptosis, signal transduction, inflammation and DNA damage repair [169]. Similar effects were seen in colon cancer cells, where D-limonene suppressed cell viability through the induction of apoptosis via the intrinsic pathway and the suppression of the PI3K/Akt pathway [167]. In gastric cancer cells, the mitochondrial-mediated intrinsic pathway was activated. When limonene was combined with berberine, synergistic anticancer effects were observed compared to either compound alone [170]. Light chain 3 (LC3) lipidation leading to autophagy was observed in neuroblastoma cells, and this lipidation was independent of ROS generation or ERK activation and caused a decrease in p62 levels [172,173]. Autophagy was also found in lung cancer cells lines and D-limonene suppressed tumor growth in murine models [174]. D-Limonene induced cell apoptosis in murine T-cell lymphoma cells by two mechanisms. At low concentrations, it resulted in production of H_2_O_2_ and ERK pathway activation, while at high concentrations it inhibited the farnesylation of proteins and O_2_ production [179]. Niosomes containing D-limonene (20 µM) exerted cytotoxic effects on HepG2 and other cell lines [168]. The combination of D-limonene and docetaxel improved the cytotoxicity to DU-145 human prostate carcinoma cells compared to docetaxel alone. This combination resulted in increased ROS production and apoptotic protein expression, suggesting the mitochondrial pathway of apoptosis was implicated [171].

Several studies have shown the in vivo anticancer effects of dietary D-limonene ranging from 7.5–10 percent on mammary carcinoma models. D-limonene increased the latency period for these tumors, caused regression, and inhibited subsequent tumor formation. This was shown in both small spontaneously regressing and large non-spontaneously regressing mammary tumors in rats [175,176,177]. In humans, early stage breast cancer patients receiving 2 g of limonene daily had a change in metabolites present in their tumors after surgical resection. These changes were correlated with a decrease in cell cycle regulatory proteins such as cyclin D1 [186]. Other studies have found that D-limonene treatment of induced hepatocarcinogenesis in mice reduced transcription of the oncogenes c-jun and c-myc [184]. D-limonene decreased the incidence and metastasis of gastric cancers through the induction of apoptosis and decreased DNA synthesis, and inhibited pulmonary adenoma formation in mice [180,181,182]. In Syrian golden hamsters, D-limonene inhibited the development of pancreatic carcinoma, however, it did not induce tumor cell loss through apoptosis, rather it inhibited cell proliferation [178]. Raphael and Kuttan [185] demonstrated that limonene reduced metastatic tumour nodule formation in a murine melanoma model. Chaudhary et al. [183] further noted that D-limonene treatment reduced tumor incidence and burden, and extended the latency period of tumor development in a murine skin cancer model. This was potentially mediated through the induction of apoptosis, inhibition of inflammation, oxidative stress, and ras-signalling [183].

### 3.5. Pinene

There are two structural isomers of the bicyclic monoterpene pinene: α-pinene and β-pinene. Pinene is an important constituent of pine resin, where it gives the pine tree aroma, but is also found in the resins of many other conifers, as well as in some non-coniferous plants like rosemary, dill, basil, and parsley. Both isomers are part of the chemical communication system of many insects. It is also the major constituent of turpentine. α-pinene reduced cell viability in human ovary cells. It achieved this through caspase-3-mediated apoptosis [187]. Similarly, proliferation of HepG2 hepatocellular carcinoma cells was inhibited by α-pinene treatment and resulted in increased ROS production, leading to apoptosis [188]. Several studies by Chen et al. [193,194] have shown that α-pinene induces cell cycle arrest regulated by cell cycle checkpoint kinases Chk1 and Chk2 in hepatoma cells. The inhibition of cell cycle transition between the G2 and M phase shown by Chen et al. was also seen in PA-1 and HepG2 cells [187,188]. α-pinene inhibited PC-3 human prostate cancer cell growth, induced apoptosis, and reduced tumor progression in mice with xenograft tumors [189]. In melanoma cells, α-pinene increased ROS production and early apoptotic features such as DNA fragmentation and phosphatidylserine on the cell surface, as well as disruption of the mitochondrial membrane potential. α-pinene treatment also reduced the number of lung tumor nodules in mice [191]. Both α- and β-pinene have been shown to act synergistically with paclitaxel in A549 lung cancer cells. α-pinene treatment combined with paclitaxel increased cells in G0/G1 stage of the cell cycle and decreased the amount of sub G0/G1 cells. The combination of both α-pinene or β-pinene with paclitaxel induced nuclear morphological changes that are characteristic of apoptosis [192]. Interestingly, an essential oil containing (−)-β-pinene and (+)-β-pinene, among other terpenes, was anti-proliferative against MCF-7, A375 and HepG2 cells. However, when the cells were treated with each compound individually, (−)-β-pinene and (+)-β-pinene showed very weak anti-proliferative effects, suggesting there may be synergistic effects happening within the whole essential oil [190].

### 3.6. Linalool

Linalool is a monoterpene that is common to numerous plants like coriander and bay laurel, with a characteristic lavender aroma with a hint of spiciness. Oral cancer cells treated with 10 µM linalool displayed reduced viability as a result of apoptosis and sub-G1 cell cycle arrest, along with a decrease in p-AKT and PI3K expression in a concentration-dependent manner [195]. Similarly, linalool (0–2.5 mM) inhibited HepG2 cell proliferation through G0/G1 cell cycle arrest and apoptosis using signaling pathways involving Ras MAPKs and Akt/mTOR [196]. In glioma cells, linalool concentrations ranging from 25–100 µM reduced cell viability and induced apoptosis via increased expression of Bax, Bak, caspase-3, and caspase-9 and decreased expression of Bcl-2 and Bcl-xl [197]. Linalool’s anti-proliferative effects in lymphoma (U937), cervical (HeLa), breast, colorectal, and liver cancer cells were also observed, again with sub G1 cell cycle arrest, while HeLa cells arrested at the G2/M phase [198,199]. In vivo studies have shown that linalool was effective in reducing tumor size in mouse models. One study has shown that treatment with (200 mg/kg) linalool reduced xenograft tumor weight by 55 percent and caused tumor specific lipid peroxidation. Delayed lipid peroxidation, leading to apoptosis, was also shown by the same group in vitro [200]. Linalool reduced murine sarcoma tumor volume via mechanisms involving oxidative stress, however, did not exhibit hepatotoxicity or myelosuppression in vivo and only exerted pro-oxidant effects in tumor tissue. Similarly, oxidative stress was implicated in the apoptosis seen in S-180 sarcoma cancer cells [201]. Interestingly, treatment with linalool prior to chronic UVB-exposure in mice reduced tumor incidence and expression of proliferative markers in mouse skin. Linalool may act as a chemo-preventive agent by inhibiting the development of dysplasia and squamous cell carcinoma (SCC) in the chronic UVB-exposed mouse skin model [204]. Linalool also acted synergistically with other chemotherapeutic agents, for example, the combination of linalool and doxorubicin increased the doxorubicin influx system, particularly through the Na^+^-dependent nucleoside transporter 3, causing increased cytotoxic doxorubicin effects in leukemia cells. Additionally, the combination of linalool with doxorubicin decreased tumor weight in male BDF1 mice compared to doxorubicin treatment alone [202]. Similar synergy between linalool and doxorubicin was also observed in multidrug resistant MCF-7 breast cancer cells, where linalool increased doxorubicin accumulation and a decrease in Bcl-xl expression was seen [203].

### 3.7. Bisabolol

Bisabolol, a monocyclic sesquiterpene alcohol, is described as having a sweet and floral aroma reminiscent of chamomile. α-bisabolol was shown to induce cytotoxicity in transformed cells, while deprived of general toxicity in several mouse models [205]. The inhibitory effects of bisabolol have been shown in various types of cancer; non-small cell lung carcinoma cells (IC_50_ of 15 μM) [206], human and rat glioma cells (IC_50_ between 2.5–5 μM and 45 μM depending on the report) [207,208], B-chronic lymphocytic leukemia (IC_50_ 42 μM) [209], as well as several other cancers such as primary lymphoid leukemias, pancreatic cancer cell lines, PC-3, HeLa, ECA-109 and HepG2 [210,211,212]. In vivo, 10 mg decreased the number of the palpable tumor masses in a mammary tumor model in HER-2/neu transgenic mice [218]. A derivative of α-bisabolol (derivative 5) was also shown to inhibit xenograft tumor growth and reduce the spread of pancreatic cancer to peritoneal nodules [219]. Some reports suggest that α-bisabolol could inhibit the PI3K/AKT signaling pathway in a dose-dependent manner, induce the activation of both caspase-dependent and independent cell death pathways, autophagy, and apoptosis [206,217].

Radiotherapy is widely used against endometrial cancer, and α-bisabolol improved the sensitivity of endometrial cancer cells to radiotherapy and further inhibited the growth of endometrial cancer cells [213]. In oral cancer, 5-aminolevulinic (5-ALA) acid is used in photodynamic therapy but exhibits poor penetration of oral tissues due to its high hydrophilicity. α-bisabolol improved 5-ALA retention in buccal tissues (6-fold higher than 5-ALA alone) where a mixture containing 1 percent 5-ALA and 5 percent α-bisabolol provided the best results [220]. Finally, while chamomile extracts do not solely contain bisabolol and are also rich in cytotoxic compounds that could mediate effects, bisabolol-rich chamomile extracts inhibited the growth of breast, ovarian, skin, and prostate cancer cells [214,215,309].

### 3.8. Elemene

The elemenes, including α-, β-, γ-, and δ-elemene, are structural isomers and are classified as sesquiterpenes. β-elemene can be isolated from various plants including the Chinese medicinal herb *Rhizoma Zedoariae* and has a fresh, herbal, and waxy taste. It has been explored as a potent anti-cancer agent against multiple cancers in several small clinical trials as well as in experimental research in vivo and in vitro, where it showed antiproliferative effects via cell-cycle arrest and induction of apoptosis [216,221,222,223,224,225]. It also enhanced the activity of chemotherapeutic agents or other conventional treatment methods in various cancer types [227,228,229,230,231]. β-elemene exerts therapeutic potential via modulation of core hallmarks of cancer. Elemene has been shown to suppress signaling such as MAPK and PI3K/Akt/mTOR pathway involved in proliferation, induce cell death, up-regulate growth suppressors, deactivate invasion and metastasis, and attenuate angiogenesis (reviewed in [226]). Large variations in the quality and size of clinical trials reduces the reliability of the interpretation of the results, but a meta-analysis of 38 clinical trials showed an overall positive effect of elemene in combination with chemotherapy in lung cancer, hepatocarcinoma, metastatic brain cancer, and leukemia, but not in gastric carcinoma [235]. Another meta-analysis identified elemene injection as a safe and effective adjunctive treatment to platinum-based chemotherapy in patients with stage III/IV non-small cell lung cancer, where it showed enhanced efficacy and cellular immune function, as well as reduced the toxicity of chemotherapy. However, it was also noted that further randomized clinical trials with significant survival outcomes and longer follow-ups are required to confirm the results further [234]. Interestingly, it was also shown to reverse multidrug resistance. Elemene increased the cytotoxicity of paclitaxel, colchicine, and vinblastine by inhibiting the efflux activity of the ABCB1 transporter [232,233]. While concentrations of elemene used would be unlikely to be found in cannabis extracts, the possibility of synergy of elemene with chemotherapeutic phytocannabinoids should be explored in therapeutic formulations.

### 3.9. Eudesmols

Eudesmol are sesquiterpenes. β-eudesmol can be found in walnut, sweet basil, ginkgo nuts, and burdock. One study found that all eudesmol isomers had cytotoxic effects in many tumour cell lines [236]. β-eudesmol also inhibited human lung and colon cancer cell lines proliferation, inhibited superoxide production in lung cancer cells and inhibited adhesion and migration some of the cell lines studied [238]. A high dose β-eudesmol treatment (10–100 μM) inhibited proliferation of HeLa, SGC-7901, and BEL-7402 tumour cells in a time- and dose-dependent manner, while a low dose β-eudesmol treatment (2.5–5 mg/kg) significantly reduced tumour growth in vivo [237]. Other studies assessed the anti-cholangiocarcinoma activity and pharmacokinetics of β-eudesmol in vitro, in a cholangiocarcinoma-xenografted nude mouse model and in healthy mice. β-eudesmol exerted significant cell growth inhibition, cell cycle arrest, and apoptosis in cell lines (IC_50_ 39 μg/mL) [239,240]. Tumour size and lung metastasis were significantly inhibited following treatment with high-dose β-eudesmol (100 mg/kg body weight for 30 days) and survival time was increased [242]. β-eudesmol also potentiated the cytotoxic and inhibitory effects of 5-fluorouracil and doxorubicin on cell migration in a cholangiocarcinoma model [241].

### 3.10. Eucalyptol

Eucalyptol, also known as 1,8-cineole, gets its name from Eucalyptus, as it is the major constituent of eucalyptus oil. Wormwood, rosemary, and sage also contain this compound. Eucalyptol is a cyclic ether and monoterpenoid with a fresh, minty aroma. Eucalyptol was shown to induce apoptosis in several human cancer cell lines, including leukemia cells. The formation of apoptotic bodies and DNA fragmentation was observed after treatment, and this was both time- and concentration-dependent [244]. Cytotoxic effects and pro-apoptotic characteristics were also observed in human ovarian cancer cells. Treatment with up to 1 µM eucalyptol caused a dose-dependent increase in early apoptosis, while limiting necrosis. In addition, eucalyptol caused S phase arrest and dose-dependent increase in pre-G1 apoptotic events was also observed [245]. Eucalyptol induced cell death in prostate cancer cells expressing the tumor suppressor gene ANXA7. Synergistic effects between eucalyptol and ANXA7 caused changes in gene expression of genes including the Ras family, MDM4, NF-ĸB and VEGF [243]. Additionally, eucalyptol caused inactivation of survivin and Akt, and activated p38 in human colon cancer cells, leading to increased cleaved PARP and caspase-3 and induction of apoptosis. In SCID mice xenotransplanted with colon cancer cells, eucalyptol-treated mice showed significantly reduced tumor progression compared to the control group [246]. In human epidermoid carcinoma cells, 10–30 µg/mL of eucalyptol led to apoptosis and G2/M phase cell cycle arrest through increased expression of p53, Bax/Bcl-2, Cytochrome c, caspase-9, and caspase-3 following treatment. Molecular docking simulations revealed the interaction of eucalyptol with Bcl-2 and PARP1 receptor [247].

### 3.11. Borneol

Borneol is a bicyclic organic compound and a terpene derivative. Borneol, like eucalyptol, has a distinctively camphor-like but slightly earthier aroma. Few studies have reported direct effects of borneol related to effects as an anticancer drug, although synergism with other drugs has been observed. Borneol inhibits P-glycoprotein efflux pumps, often involved in cancer resistance of chemotherapeutic drugs. In one study, borneol chemo-sensitized human glioma cells to doxorubicin, where it increased doxorubicin-induced G2/M cell cycle arrest through ROS-mediated DNA damage. DOX-induced interference with MAPKs and PI3K/AKT pathways was also increased, and these effects were repeated in a human glioma xenograft model [248]. Borneol also effectively synergized with paclitaxel to inhibit the survival of esophageal squamous cell carcinoma (ESCC) cells by inducing apoptosis through suppression of the PI3K/AKT pathway [249]. Similar effects were seen with selenocystine in human hepatocellular carcinoma cells, where enhanced cellular uptake of selenocystine occurred [250]. Synergy of borneol was also observed with curcumin-based drugs in human melanoma cells and HepG2 cells [251,252], and temozolomide in glioma tumours [253].

Borneol is reported as a ‘guide’ drug in traditional Chinese medicine and has been shown to promote passage of free drugs across the blood–brain barrier (BBB) efficiently. Borneol can induce transient disruption of the BBB after 20 min of oral administration [259]. Based on these observations, borneol has been used in a number of studies as a part of a drug formulation to target cancer cells in the brain. The borneol combination could further enhance the anti-tumour efficiency of multiple drug targeting systems after penetrating the BBB. These effects were observed with a borneol-modified nanomicelle delivery system with doxorubicin for glioblastoma therapy in vitro and in vivo [254]. Similar effects were obtained using PEG-PAMAM nanoparticles co-loaded with paclitaxel and borneol in ovarian cancer cells [255]. Other nanomolecule formulations have also shown synergistic effects when combined with borneol [256,257,258].

### 3.12. Terpineol

Terpineol can refer to any combination of four monoterpene isomers. α-terpineol is the most common in cannabis. The lilac flower aroma of this terpene is often found in plants that also contain high concentrations of pinene. Various essential oils containing terpineol showed cytotoxic effects. For example, glioblastoma cells were sensitive to an essential oil extract from *Ocimum basilicum*, where the major constituents were α-terpineol (59.78%) and β-caryophyllene (10.54%) [260]. α-terpineol also displayed antiproliferative effects on human erythroleukemic cells [262]. γ-terpineol treatment significantly suppressed human hepatoma cell proliferation in a dose-dependent manner, and induced changes characteristic of apoptosis. Accumulation of cells at G1 or S phase and a reduction in proliferation were also noted [263]. The isomer 4-terpineol induced dose-dependent cytotoxicity in hepatocellular carcinoma (HCC) cells. Treatments up to 100 μM resulted in inhibition of wound healing–a measure for cell migration–in a dose-dependent manner. HCC cells treated with 4-terpineol accumulated in the sub-G1 phase of the cell cycle. In vivo, 10 and 20 mg/kg of 4-terpineol decreased the tumor weight and tumor volume in a dose-dependent manner [261]. Finally, α-terpineol potentiated the cytotoxic effects induced by oxaliplatin and 5-fluorouracil in a colon cancer cell line [264].

### 3.13. Terpinene Isomers

Terpinenes are classified as monoterpenes. α-terpinene can be found in cardamom and marjoram oils and displays an aroma described as woody with hints of pine, citrus and spicy mint. γ-terpinene has been isolated from a variety of plant sources, such as citrus, and displays a woody, lemon-lime scent. δ-terpinene (also known as terpinolene) can also be found in sage, rosemary, apples, tea trees, cumin and nutmeg. It also exudes woodsy aroma with citrus and floral notes. β-terpinene has not been found in a natural source. Studies showed that some essential oils containing γ-terpinene exhibited antiproliferative properties in multiple cell lines, including MCF-7, 4T1, HepG2, Jurkat, and HeLa cancer cells. The most commonly proposed mechanism of action for the antiproliferative effects was the induction of apoptosis [265,266,267,268]. Interestingly, in studies that further investigated the components of the essential oils individually, they found that γ-terpinene was not responsible for the antiproliferative effects demonstrated by the whole essential oil extract [262,267].

### 3.14. Valencene

Valencene is a sesquiterpene with a citrus-like aroma and is commonly found in Valencia oranges, as well as grapefruits, nectarines, mangoes, and tangerines. Valencene was shown to display cytotoxic and anti-proliferative effects in various cancer types, including ovarian cancer cell lines (doxorubicin-sensitive A2780 and partially resistant SKOV3) and lymphoblast cancer cell lines (doxorubicin-sensitive CCRF/CEM and completely resistant CEM/ADR) [158]. Valencene also inhibited proliferation of CaCo-2 cancer cells and acted synergistically with doxorubicin [269]. In contrast, valencene was ineffective at enhancing the anti-proliferative effects of 5-fluorouracil and oxaliplatin in cancer cell lines Caco-2 and SW-620, while other terpenes like humulene and caryophyllene oxide could [160].

### 3.15. Geraniol

Interestingly, varieties that have high levels of the terpene linalool tend to be high in geraniol as well. Geraniol occurs naturally in plants like geraniums, roses, peaches, lemongrass, coriander, lemons, and is even produced by the scent glands of honeybees, where it serves as a mark of nectar-bearing flowers and a guide for the bees to locate the entrance to their hives [310]. Geraniol had anticancer effects in many types of cancers, including breast, lung, colon, prostate, pancreatic, skin, liver, kidney and oral cancers. A detailed publication by Cho and al. [311] reviews geraniol’s effects on cancer hallmarks for a variety of cancer types. Thus, we complement this previous review with the more recent advances regarding the effects of geraniol in cancer since its publication. One study examined the effects of geraniol in endometrial carcinoma in rats. They showed that geraniol exhibited anticancer effects via downregulation of oncogenes and upregulation of tumour suppressor genes, acting via MAPK pathways and Wnt signaling pathways [312]. Geraniol also showed antiproliferative and pro-apoptotic effects on hepatocellular carcinoma cell lines SMMC7721 and HepG2. A suggested mechanism for the effects of geraniol was the decreased expression of Bcl-2 and upregulation of Bax and caspase [270]. Anticancer effects by geraniol and geranyl acetate were also found in cancer cell lines, with IC_50_ values of 20 and 30 μM respectively. Similar to what was found in the hepatocellular carcinoma cell lines, upregulation of Bax and downregulation of Bcl-2 expressions, leading to intrinsic apoptosis were shown. DNA damage and G2/M cell cycle arrest in COLO-205 colon adenocarcinoma cells was also observed [271]. In prostate cancer, experiments demonstrated that geraniol down-regulated E2F8 expression sufficiently to suppress cell growth by inducing G2/M arrest [272].

### 3.16. Nerolidol

Nerolidol, a sesquiterpene alcohol, is also known as peruviol and penetrol. It is found in the essential oils of many types of plants and flowers including neroli, ginger, jasmine, lavender, tea tree, lemongrass and is a dominant scent compound in *Brassavola nodosa*, a type of orchid [313]. The aroma of nerolidol is reminiscent of fresh bark. Nerolidol has been shown to have some anticancer effects in HeLa cervical cancer cells, Jurkat leukemia cells and breast carcinoma cells at concentrations less than 5 µM [273,274]. A later study noted that cis-nerolidol possessed strong cytotoxic properties in HeLa cells at 16.5 µM [275]. In leukemia cells, the inhibition of growth was attributed to alterations in the cell cycle as well as increases in the proportion of apoptotic cells. Nerolidol caused dose-dependent increases in the proportion of cells in the G0-G1 phase and decreases in the proportion of cells in the S phase [276]. Trans-nerolidol exhibited anticancer effects in colorectal cancer cells through the induction of apoptosis in the presence of tumor necrosis factor (TNF) α. These apoptotic effects were mediated through increases in caspase activity and decreases in phosphorylation of NF-κB. Additionally, trans-nerolidol significantly decreased adhesion of TNFα-induced cells, likely through the down-regulation of ICAM-1 [159]. In vivo, it has been shown that a daily diet of nerolidol (5 mg/g) had an inhibitory effect on azoxymethane-induced cancer of the large bowel and duodenum in male rats. Nerolidol both reduced the number of rats presenting with neoplasms of the large bowel and decreased the number of tumors per rat [277]. Two important studies have noted the potential of nerolidol when combined with the anticancer drug doxorubicin. In one study, nerolidol inhibited proliferation of CaCo-2 colon cancer cells on its own while leaving non-cancerous hepatocytes unaffected. When combined with doxorubicin, nerolidol was able to synergistically exacerbate the effects of doxorubicin by increasing its accumulation in CaCo-2 cells, while not altering its accumulation in normal hepatocytes [269]. Nerolidol also acted synergistically with doxorubicin to reduce viability in ovarian cancer cells and lymphoblast cells at high concentrations (100–200 µM) [159].

### 3.17. Guaiol

The aromas associated with guaiol in cannabis are woodsy, floral or rosy. According to Lawless, guaiol, a bicyclic sesquiterpenoid alkene alcohol, is a major component (42–72%) of the essential oil of guaiac wood from the species *Bulnesia sarmientoi* with a pleasant rose-like aroma [314]. Studies have shown that extracts from *Bulnesia sarmientoi* displayed anti-proliferative and anti-metastatic effects in lung cancer cell lines. α-guaiene, (−)-guaiol and β-caryophyllene were suggested as the mediators for most of the cytotoxic activity of the extract against two cancer cell lines [278]. In a series of studies from the same group, (−)-guaiol was shown to significantly inhibit cell growth of non-small-cell lung carcinoma (NSCLC) cells both in vitro and in vivo. Levels of RAD51, involved in repair of DNA double strand breaks, were implicated in the chemosensitivity of NSCLC cells to (−)-guaiol both in vitro and in vivo [279]. It was also suggested that in NSCLC cells, (−)-guaiol significantly blocked the mTORC2-AKT signaling by inhibiting mTOR phosphorylation to induce autophagy [280]. Finally, guaiol was also shown to inhibit NSCLC cells in vitro, and in vivo in nude mice with an efficacy similar to cisplatin when the same dose of each drug was administered (8 mg/kg) [279].

### 3.18. Camphene

Camphene is a bicyclic monoterpene that can be found as a minor constituent of several essential oils from cypress oil, citronella oil, camphor oil, ginger oil, as well as other plants, such as neroli and valerian. Similar to myrcene, camphene has an earthy aroma reminiscent of fir needles. Camphene inhibited proliferation in several cancer cell lines, such as B16F10-Nex2, A2058, HeLa, HL-60, U87-MG, and SKBR-3 cells with IC_50_ values ranging from 10–71 µg/mL. In B16F10-Nex2 melanoma cells, camphene caused endoplasmic reticulum stress, loss of mitochondrial membrane potential, and upregulation of caspase-3, resulting in apoptosis. Additionally, camphene had antitumor activity in vivo by reducing subcutaneous tumor growth of melanoma cells in a syngeneic model [281].

### 3.19. Alpha-Phellandrene

α-phellandrene and β-phellandrene are cyclic monoterpenes. α-phellandrene was named after *Eucalyptus phellandra*, now called *Eucalyptus radiata*. Similar to eucalyptus, α-phellandrene has a citrus, minty aroma with a hint of black pepper or spice. α-phellandrene has been shown to display some anticancer effects in various models. For example, alterations in the expression genes associated with DNA damage, cell cycle and apoptotic cell death in murine leukemia cells were observed following treatment with 10 µM α-phellandrene [283]. Expression of phosphorylated-p53, phosphorylated-H2A.X, 14-3-3-σ, and MDC1 were increased after treatment with α-phellandrene, however p53, MGMT, DNA-PK, and BRCA-1 were decreased [284]. Treatment with α-phellandrene induced G0/G1 cell cycle arrest, increased reactive oxygen species production and Ca^2+^, and decreased levels of mitochondrial membrane potential, all in a dose- and time-dependent manner [286]. α-phellandrene also significantly inhibited cell viability of liver tumor cells at a concentration of 30 µM. This inhibition of cell viability was due to necrosis caused by increased nitric oxide, reactive oxygen species production and lactate dehydrogenase leakage, ultimately leading to ATP depletion [282]. A subsequent study by this group found that treatment with α-phellandrene also induced autophagy via downregulation of PI3K-I, mTOR, and Akt and upregulation of phosphorylated Bcl-2, PI3K-III, LC3-II and Beclin-1. Treatment with α-phellandrene also up-regulated nuclear p53 and activated the NF-κB pathway, further leading to necrosis [285].

### 3.20. Delta-3-Carene

δ-3-carene is a bicyclic monoterpenoid alkene that is predominantly associated with turpentine from conifers, but is also prevalent in white pepper (*Piper nigrum*, 25%) [315], and can be found in low concentrations in cannabis. Little has been reported regarding the potential effects of this compound in cancer. The cytotoxic activity of essential oils from needles and twigs of different varieties of pines where δ-3-carene can be found were determined on cell lines HeLa, CaCo-2 and MCF-7. Essential oils showed significant cytotoxic effects on the aforementioned cancer cell lines [287]. Similarly, in the essential oil from *Boswellia dalzielii*, 50 compounds were identified, including 3-carene (27.72% of essential oil composition) and α-pinene (15.18%). At 50 mg/L, extracts that differed in the solvent used for extraction of the compounds inhibited OVCAR-3 or IGROV-1 cell viability [288]. While the results of these studies did not demonstrate the effects of δ-3-carene as an anticancer drug since several other natural drugs were present in the extracts, some cytotoxicity was found with the extracts, which suggests more studies with individual drugs should be performed.

### 3.21. Cadinenes

Cadinenes are bicyclic sesquiterpenes and either display herbal, woody, or smoky aromas, and their name is derived from the Cade juniper (*Juniperus oxycedrus* L.), which cadinene isomers were first isolated from. Several species of termites and beetles use γ-cadinene in their chemical communication system [316]. A variety of essential oils containing δ- or γ-cadinene have exhibited some cytotoxic effects in various cancer cell lines [289,290,291]. Unfortunately, very few studies have used purified cadinenes to evaluate their effects as anticancer agents. The only study we found highlighted the antiproliferative and apoptotic effects of δ-cadinene on human ovary cancer cells. δ-cadinene induced dose and time-dependent growth inhibitory effects on OVCAR-3 cells. Characteristics of apoptosis, such as cell shrinkage, nuclear membrane rupture and chromatin condensation were also observed. In addition, treatment with varying concentrations of δ-cadinene ranging from 10–100 µM resulted in cell cycle arrest at the sub-G1 phase [292].

### 3.22. Thujone

Thujone is a monoterpene compound that is present in two forms: (−)-α-thujone and (+)-β-thujone. α-thujone is approximately 5 times more toxic and biologically active. It can be found in *Artemisia absinthium*, an infamous plant used in medicine and for psychotropic experiences. It is also present in multiple other plant species like Tansy, Western red cedar and sage and its aroma resembles that of menthol. This compound is a GABA_A_ receptor and 5-HT_3_ antagonist, and it displays brain, liver and kidney toxicity at higher doses [294]. The first evidence of thujone as an anticancer drug came from a thujone-rich fraction of *Thuja occidentalis*, where it showed cytotoxicity, anti-proliferative and apoptotic effects in vitro on melanoma A375 cells. Thujone induced inter-nucleosomal DNA fragmentation, mitochondrial transmembrane potential collapse, ROS generation, release of cytochrome c and caspase-3 activation [293]. Similar effects were observed using an α/β-thujone fraction from *Thuja occidentalis*, where potent in vitro anti-proliferative, pro-apoptotic and anti-angiogenic effects were observed in glioblastoma cells. Another study also showed the effects of α-thujone on the viability and proliferation of glioblastoma multiforme cells when administered at high concentrations (660 μM–3.2 mM) [317]. In vivo work demonstrated α/β-thujone’s ability to promote the regression of neoplasia [318]. α/β-thujone sensitized cells to the effects of paclitaxel in choriocarcinoma cells [319]. In vivo, administration of thujone (1 mg/kg body weight) acted prophylactically and simultaneously with tumor induction to inhibit tumor nodule formation in the lungs and increase the survival rate of animals bearing metastatic tumors. In another metastatic animal model, thujone suppressed lung metastasis of murine melanoma B16F-10 cells via inhibiting cancer cell proliferation, adhesion and invasion, and by regulating the expression of MMPs, ERK-1, ERK-2, VEGF, TIMPs, nm23 and concentrations of pro-inflammatory cytokines and interleukin IL-2 [320]. In contrast, higher doses of thujone (12.5–50 mg/kg) resulted in increased incidence of preputial gland cancers, and a minor increase in the incidence of pheochromocytomas of the adrenal gland was found in male rats, while 50 mg/kg was lethal [321].

### 3.23. p-Cymene

*p*-Cymene, a naturally occurring monoterpene, has been characterized by some as exuding a flavor profile of orange or carrot, while others perceive the terpene as a combination of aged wood and lemon in cannabis. In addition to its presence in specific cannabis cultivars, cymene can be found in over 100 different plants, including cumin and thyme. While not much is known about the effects of cymene alone, essential oils from *Nigella sativa*—which contain the major ingredients thymoquinone (up to 50%), *p*-Cymene (40%) and pinene (up to 15%)—have exhibited antitumoral effects against many cancers, including blood, renal, lung, prostate, liver, breast and other malignant cell lines [295]. *p*-Cymene is a common ligand for ruthenium, proposed as a potential alternative to platinum-based chemotherapies. Ruthenium^II^(*p*-Cymene) complexes have been suggested as effective and selective anticancer candidates under various forms for different cancer types [296,297,298].

### 3.24. Gurjunene

Gurjunene is a tricyclic sesquiterpene alkene, with a woody balsamic scent. It has been reported in cannabis, but it is challenging to differentiate it analytically from nerolidol [322]. While little is known about the anticancer effects of gurjunene as a pure compound, some studies have shown the anticancer activity of oils that contain it from plant extracts. For example, *Melicope denhamii* leaf oil–which contains terpenes zierone (22.49%) and α-gurjunene (19.96%) as the major components–displayed anticancer activity against Dalton’s lymphoma ascites cells via induction of apoptosis [323]. The essential oil from the leaves of *Annona sylvatica*, in which γ-gurjunene is present, showed inhibitory effects on growth in all cell lines tested when administered at high concentrations (GI_50_ values ranging from 36–45 μg/mL on all of the cell lines tested) [299]. Using *Dipterocarpus alatus* extracts (a medicinal plant used for the treatment of genito-urinary diseases) where the major components were α-gurjunene (30.31%), (−)-isoledene (13.69%), alloaromadendrene (3.28%), β-caryophyllene (3.14%) and γ-gurjunene (3.14%), cytotoxic activity of the oleo-resin was credited to its sesquiterpene content [300]. While none of these studies were done with pure compounds, they do suggest some potential anticancer activity by gurjunene compounds. More studies are required to determine whether gurjunene could be useful in cancer therapy and in which capacity.

### 3.25. Farnesene

Two types of farnesene can be found naturally. Farnesene is found in gardenia, apples and several other fruits (often in the rinds of the fruit) as well as ginger, nutmeg and basil. α-farnesene present in the skin of green apples gives it its aroma. β-farnesene has been described as a woody aroma. Aphids and other insects can release farnesene as an alarm pheromone [301,324]. Few studies have evaluated the effects of farnesene in cancer, but some plant extracts containing farnesene have been studied. *Garcinia atroviridis* leaf oil is rich in (E)-β-farnesene (58.5%) and β-caryophyllene (16.9%). Treatment of MCF-7 cells using the leaf oil at 100 μg/mL induced cell death and acted synergistically with tamoxifen [302]. The essential oil from *Cedrelopsis grevei* leaves, rich in (E)-β-farnesene (27.61%) and δ-cadinene (14.48%), exhibited cytotoxic effects on MCF-7 cells (IC_50_ of 21.5 mg/L) [303]. Finally, an essential oil from the leaves of *Panax ginseng* C.A. Meyer where the major components were palmitic acid (36.1%), β-farnesene (15.4%), and linoleic acid (9.8%), exhibited cytotoxic actions against a variety of cancer cell lines, including HeLa, A549, ZR-75-1, HT-29, SGC7901 and B16 cells [304]. Similar to other lesser characterized terpenes, more studies are required using pure compounds to truly assess a potential role of farnesene in cancer therapy.

## 4. Flavonoids

**Figure 3:**
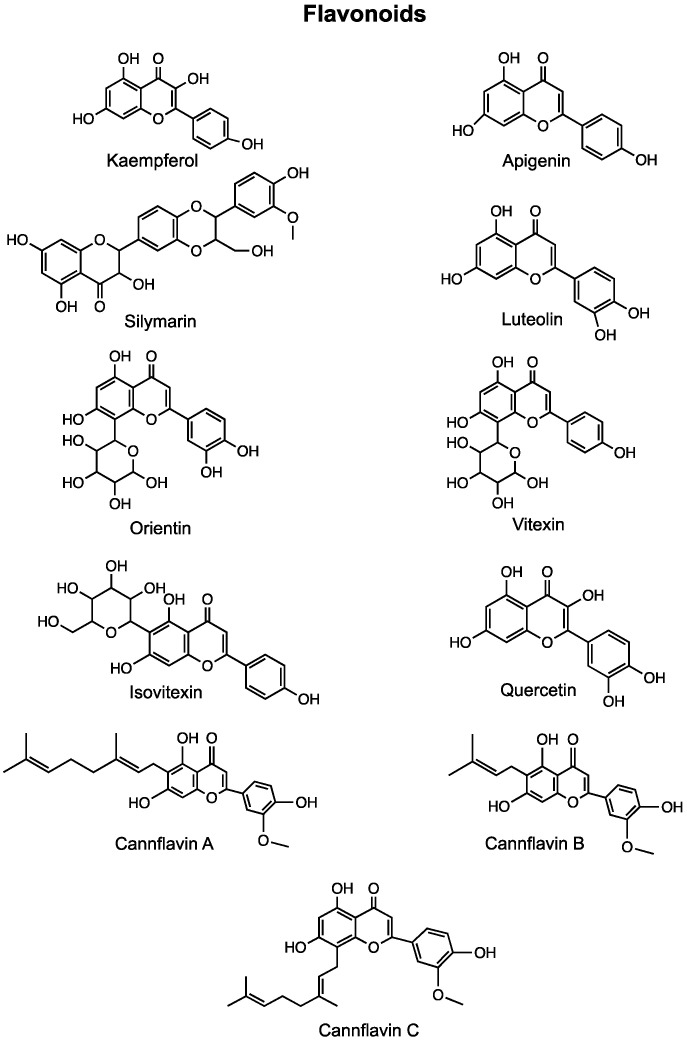
Structure of various flavonoids found in the Cannabis plant.

Flavonoids are by far the largest class of polyphenols and have been estimated to contain over 8000 metabolites with vast structural and functional diversity. Flavonoids have been subdivided into six major subclasses: flavones, flavonols, flavanones, flavanols, isoflavones and anthocyanidins [325]. One of their properties is to provide the vivid color pigmentation in flowers, fruits and vegetables. Flavonoids account for roughly 10 percent of compounds known in cannabis, with around 20 types known in the cannabis plant, mainly belonging to the flavone and flavonol subclasses [326]. Some of the best-known flavonoids are quercetin and kaempferol and some are uniquely found in cannabis, such as the cannflavins (Figure 3). The distribution of the flavonoids in the cannabis plant varies; while they are quasi non-existent in seeds and roots, they may represent up to 2.5 percent of cannabis’ leaf and flower dry weight. While the effects of flavonoids have been studied in multiple other plants, little is known about their potential effects in regard to their interactions with other compounds present in cannabis and therapeutic effects in various aspects of cancer. We provide here an overview of the effects identified for the main flavonoids present in cannabis, related to cancer (summarized in Table 3).

### 4.1. Kaempferol

Kaempferol is a well-characterized natural flavonol that is commonly found in dietary items like tea, apples, strawberries, broccoli, and beans [327]. It is also produced by the *Cannabis* plant and has attracted much research surrounding its potential health benefits, including its potential as an anti-cancer agent. A plethora of research in recent years has demonstrated many of kaempferol’s anti-cancer effects in vitro and in vivo on a variety of cancer subtypes. Two comprehensive reviews by Irman et al. [328] and Kashyap et al. [327] discussed the anti-cancer effects of kaempferol in various cancers. Here we highlight the main review findings and discuss more recent studies that looked at kaempferol’s potential as an anti-cancer agent. Kaempferol treatment inhibited cell viability in a dose-dependent manner in a multitude of cancer subtypes. Most of the studies reviewed indicated that kaempferol’s inhibitory effects on cell viability in cancer cells were as a result of cell cycle arrest or apoptosis. Kaempferol was able to induce cell cycle arrest at the G2/M phase in a multiple cancers, including leukemia, breast, liver, stomach and ovarian cancers [329,336,337,338]. In oral cancers, kaempferol also induced cell cycle arrest, but alternatively at the G0/G1 phase [339]. In glioblastoma, hepatic, colorectal, pancreatic, lung, renal and breast cancer cell lines, kaempferol was able to significantly reduce migration and/or invasion in vitro [341,342,343,344,345]. In lung and breast cancer cell lines (A549 and MDA-MB-231/MCF-7), studies have found that treatment with kaempferol was able to inhibit the epithelial-mesenchymal-transition (EMT), resulting in decreased metastasis and resistance to chemotherapeutics in these cells [343,347]. The detailed mechanisms of the anti-cancer actions of kaempferol in breast cancer can be found thoroughly reviewed by Wang et al. [422]. Several studies have demonstrated the ability of kaempferol to reduce angiogenesis; this has been demonstrated in ovarian cancer cells as a result of altered expression of vascular endothelial growth factor [348]. Studies investigating the anti-cancer effects of kaempferol in vivo were also reviewed by Irman et al. [328,423]. In in vivo mouse models of various cancers, including bladder, oral, prostate, lung and bone cancers, treatment with kaempferol was able to increase survival and reduce the growth and metastasis of tumours [339,352,353,354,355]. In a rat model of leukemia, treatment with kaempferol resulted in degranulation in basophilic leukemia cells (RBL-2H3) and increased the accumulation of mediators in human leukemic mast cells (HMC-1) [357,358].

In addition to the studies highlighted in the reviews mentioned previously, several studies have since been published that investigated the anti-cancer potential of kaempferol. In gastric, colon, prostate, colorectal and neuroblastoma cancer cells, kaempferol was able to significantly decreased cell viability and proliferation [330,331,332,333,334]. Kaempferol treatment induced autophagy in gastric cancer cells [330]. In breast cancer cells, kaempferol was able to induce apoptosis, cell cycle arrest at the G2/M phase, and suppress cell proliferation [329]. In a mouse model of breast cancer, kaempferol treatment was able to suppress primary tumour growth and lung metastasis [356]. In ovarian cancer cell lines, treatment with kaempferol inhibited growth with IC_50_ values between 25–50 µM [335]. Further investigation revealed that kaempferol caused cell cycle arrest at the G2/M phase and induced apoptosis in OVACAR-3 ovarian cancer cells due to upregulation of apoptotic proteins such as caspase 3 and Bax [335]. Kaempferol exhibited anti-proliferative effects in endometrial carcinoma cells through apoptosis and cell cycle arrest, and was able to decrease migration and invasion trends in these cells [340]. In immortalized human retinal pigment epithelial (ARPE-19) cells, treatment with kaempferol was able to decrease cell migration through ERK1/2 signaling [346]. Kaempferol-conjugated gold nanoclusters (K-AuNCs) were developed by Govindaraju et al. [424] as a potential anti-cancer drug delivery system, and they demonstrated that K-AuNCs targeted A549 lung cancer cells and exhibited toxicity via nucleus damage.

A few studies have investigated the potential of combination treatment with kaempferol and previously established anti-cancer agents or other compounds. A study by Seydi et al. [349] using cancerous hepatocytes from a rat model of hepatocellular carcinoma found that kaempferol combined to luteolin (another common flavonoid) was able to inhibit cell proliferation, induce cell death and inhibit migration and invasion. Tumor necrosis factor-related apoptosis-inducing ligand (TRAIL) stimulates apoptosis through binding death receptors 4 and 5 in a variety of cancers, however resistance to TRAIL has been known to occur [350]. Hassanzadeh et al. [350] found that co-treatment of lymphoblastic leukemia (MOLT-4) cells with TRAIL and kaempferol was able to induce apoptosis by inhibiting the expression of anti-apoptotic proteins and up-regulation of death receptors 4 and 5, and they suggested that this co-treatment could be used as a potential solution to overcome resistance to TRAIL in cancers. Li et al. [351] looked at the potential of combining 5-fluorouracil (5-FU) with kaempferol in colorectal cancer. They found that the combination of 5-FU and kaempferol was superior at inhibiting cell viability than either agent alone, and the anti-cancer effects were mediated through reduction in cell proliferation and induction of apoptosis. Another study looked at the combination of 5-FU with kaempferol in 5-FU-resistant colon cancer cells [341]. They found that combination treatment of 5-FU with kaempferol had synergistic effects on cell viability and was able to chemo-sensitize the resistant cells.

### 4.2. Apigenin

Apigenin is a natural flavone found in many fruits and vegetables, and predominantly found in parsley, celery, and in the flower of chamomile plants, among others. Apigenin is a pigment, yellow in color. A comprehensive review by Imran et al. [425] provides details about the anticancer effects of apigenin in various types of cancer such as breast, lung, liver, brain, skin, blood, bone, colon, prostate, pancreatic, cervical, ovarian, oral, and stomach. Detailed mechanisms of action of apigenin in each of these cancer types can be found within the review however, the induction of apoptosis, upregulation of caspases-3, -8 and TNF-α, downregulation of MMP-2, -9, NFkB, PI3K, Akt and pAkt, and modulation of kinases are mechanisms frequently involved. Since the review’s publication, further studies have investigated apigenin’s anticancer effects. Apigenin reduced cell viability in MCF-7, A549, HepG2 and normal HEK 293 cell lines with the greatest activity against HepG2 liver cancer cells (EC_50_ of 12 µg/mL) [359]. Li et al. [362] further noted that in three hepatocellular carcinoma cell lines, apigenin was cytotoxic and induced G1 phase cell cycle arrest in a dose dependent manner through the regulation of CyclinD1 and CDK4. Additionally, hypoxia-inducible factor 1α (HIF-1α) is associated with hypoxia-induced resistance in cancer cells but has been shown to be inhibited by apigenin. Two pathways involved in suppressing the HIF-1α expression in hypoxic tumors are through the inhibition of the AKT/p-AKT pathway and HSP90, which also enhance the activity of the chemotherapeutic paclitaxel. Apigenin and paclitaxel also acted synergistically in a liver cancer cell line and murine models [362]. When combined with the chemotherapeutic sorafenib, apigenin decreased cell viability of liver cancer cells to a greater extent than either drug alone. This combination caused an increase in apoptosis and decreased the migration and invasion capability of the cells [364].

In a colon cancer and lymph-endothelial cell model, treatment with apigenin reduced the formation of circular chemorepellent-induced defects in the endothelial barriers [426]. In a cervical cancer model, apigenin inhibited cell growth in vitro and tumour growth in vivo through the ER-mediated PI3K/Akt/mTOR pathway [365]. Similarly, in a model of diffuse large B-cell lymphoma, apigenin inhibited proliferation and colony formation by activating pro-apoptotic proteins, downregulating cell cycle proteins to increase G2/M phase arrest and inhibiting the PI3K/mTOR pathway. Apigenin also acted synergistically in vitro and in vivo with Abivertinib, a bruton tyrosine kinase inhibitor, which can provide new options for patients who have developed resistance to traditional therapies [360]. Apigenin altered the tumor necrosis factor and IL-10 release by microglia. When treated with conditioned medium of microglia treated with apigenin, C6 glioma cells exhibited reduced tumor migration and viability, due to the reduction in IL-6 levels. Apigenin also preferentially reduced viability of C6 glioma cells when co-cultured with microglia [363]. One study showed that apigenin inhibited cell proliferation, induced apoptosis, reduced vascular endothelial growth factor (VEGF) expression, and reduced tumor-induced angiogenesis in two human esophageal cancer models [361]. The inhibition of IL-6 transcription further potentiated these effects, suggesting that the inhibition of IL-6 transcription was how apigenin exhibited its anticancer effects in esophageal cancer cells. Similar effects were seen in a murine xenograft model [365].

### 4.3. Cannflavins

Cannflavins are a group of prenylflavonoids uniquely found in cannabis. Cannflavins A and B are formed by a derivative of luteolin, chrysoeriol [427]. Recently, a study examined the potential of cannflavin B derivatives for the treatment of pancreatic cancer. In vitro results showed an increase in apoptosis in two pancreatic cancer cell lines treated with concentrations of FBL-03G (or caflanone), the cannflavin B derivative. In vivo local and metastatic tumor progression were delayed in pancreatic cancer animal models as well as an increase in survival compared to control cohorts [366]. In 2019, caflanone was granted orphan drug status by the United States Food and Drug Administration and clinical trials with the drug were scheduled to begin as potential treatment for pancreatic cancer. Caflanone has been identified in a rare, flavonoid-rich cannabis cultivar native to Jamaica known as Black Swan. Little is known about the potential actions of Cannflavin A and Cannflavin C in cancer.

### 4.4. Silymarin

Silymarin is a flavonoid derived from milk thistle, but is also present in artichokes, cilantro, coriander, and cannabis. Silymarin consists of three phytochemicals, silybin, silidianin, and silicristin, and has a long medicinal tradition. Silybin is its most active phytochemical and is largely responsible for the effects of silymarin. A recent review by Delmas et al. [428] provided extensive details about mechanistic actions of silymarin in various models of cancer. The review highlighted silymarin’s ability to synergize with anticancer drugs, induce cell death through both the intrinsic and extrinsic pathways, cause cell cycle arrest in the G0/G1 and G2/M phase, modulate metabolizing enzymes and drug transporters which alter cellular sensitivity to chemotherapeutics, as well as several clinical trials currently in progress. More recently, in a Burkkett’s lymphoma model, silymarin induced apoptosis and caused a reduction in toll-like receptor 8 (TLR8) mRNA expression, implicating toll-like receptors in silymarin’s anticancer activity [367]. Silymarin further decreased cell viability, increased apoptosis, and changed the mitochondrial membrane potential in glioblastoma cells [368]. Silymarin reduced cell viability and diminished migration of stomach cancer cells through the induction of apoptosis, inhibition of p-ERK and activation of p-p38 and p-JNK. In vivo, silymarin at a concentration of 100 mg/kg reduced tumor volume and induced apoptosis [369]. Additionally, silymarin dose-dependently inhibited cell growth in prostate cancer cells by initiating apoptosis. After treatment, the expression of Slit Guidance Ligand 2 (SLIT2) and Roundabout Guidance Receptor 1 (ROBO1) were increased and the expression of CXCR4 was decreased [429]. Silymarin also had antiproliferative, antimetastatic and pro-apoptotic effects in a dose-dependent manner on liver cancer cells, and also acted through the Slit-2/Robo-1 pathway [370]. Co-treatment with cold atmospheric plasma (CAP) and a silymarin nanoemulsion (SN) decreased intracellular ATP levels and downregulated the *PI3K/AKT/mTOR* survival and *RAS/MEK* transcriptional pathways in melanoma cells [371]. Silibinin (30–90 μM), a main active component of silymarin, inhibited the epithelial-mesenchymal transition (EMT) in breast cancer cells. Silibinin also inhibited cell migration and increased mitochondrial fusion, which contributed to silibinin’s inhibitory effect on cell migration. Additionally, silibinin decreased ROS production, which decreased the NLRP3 inflammasome activation [372]. In silico, silymarin was shown to inhibit the proto-oncogene B-Raf (BRAF) and the smoothened gene (SMO), two targets in anticancer therapy [430]. A phase I clinical trial studied the effects of high dose silibinin (13 g daily) and found it to be well tolerated in patients with advanced prostate cancer. The most commonly seen adverse event was asymptomatic liver toxicity (hyperbilirubinemia and elevation of alanine aminotransferase) [373].

### 4.5. Luteolin

Luteolin is a flavone commonly found in several plants including broccoli, pepper, thyme, and celery. Luteolin has been used as a source of yellow dye since at least the first millennium B.C. and originally obtained from the plant *Reseda luteola*, a common weed. A recent review by Imran et al. [328] provides extensive information about the anticancer effects of luteolin in many cancers including breast, prostate, oral, lung, kidney, cervical, placental, ovarian, skin, liver, esophageal, bladder and glioblastoma. This review provides insight about mechanisms involved in luteolin’s anticancer effects ultimately leading to reductions in cell proliferation, cell survival signaling, angiogenesis, and metastasis and an increase in apoptosis in many of these types of cancers [431]. Since this review’s publication, more studies have further evaluated luteolin’s anticancer effects.

In vivo models of melanoma showed that luteolin inhibited cell growth through the extracellular matrix pathways, the oncogenic signaling pathway, and the immune response pathways, but not through ROS induction [378]. Furthermore, luteolin reduced proliferation, migration, invasion, adhesiveness, and tube forming potential in a metastatic melanoma model. HIF-1α/VEGF signaling-mediated epithelial to mesenchymal transition and angiogenesis was implicated in the anti-metastatic effects demonstrated by luteolin [379]. Luteolin caused double-strand DNA breaks and prevented nonhomologous end joining (NHEJ) and homologous recombination (HR) in a bursal lymphoma model, and additionally caused G2/M phase cell cycle arrest in BRCA-deficient cells and inhibited Poly [ADP-ribose] polymerase 1 (PARP1) [362,385].

Luteolin decreased cell viability in breast cancer cells as well as inhibited migration and invasion by decreasing the expression of matrix metalloproteinase-9 (MMP9) [378,379,432]. Apoptosis was induced through the extrinsic and intrinsic pathways and the epithelial-mesenchymal transition (EMT) was prevented. This was mediated by increased expression of miR-203, reduced Ras/Raf/MEK/ERK signaling, cell cycle arrest at the S phase, and by reducing telomerase levels through suppressing human telomerase reverse transcriptase (hTERT) expression [374]. S100 calcium-binding protein A7 (S100A7) has been implicated in the EMT, promoting metastasis, and was inhibited by luteolin through Src/Stat3 signaling in epidermoid carcinoma cells. This reduced migration and invasion of A431-III cells and decreased metastasis in a xenograft zebrafish model [384]. Similarly, miRNA-301-3p was downregulated in pancreatic cancer cells following treatment with luteolin, causing a decrease in cell growth [380]. In several ovarian cancer cell lines, luteolin induced apoptosis through the extrinsic and intrinsic pathways. The cell cycle was disrupted and cell invasion on the collagen was altered [375]. In non-small cell lung carcinoma, luteolin induced G2/M cell cycle arrest and reduced EMT by reducing the expression of absent in melanoma 2 (AIM2), leading to decreased AIM2 inflammasome activation which was also seen in lung cancer mouse xenograft models [376]. Masraksa et al. [389] reported that luteolin showed no cytotoxic activity on lung cancer cells up to 40 µM, however 20–40 µM was able to reduce migration, invasion and the formation of filopodia in a concentration dependent manner.

Luteolin has also had various anticancer effects in colon cancer cells. Luteolin reduced the viability and proliferation of colon cancer cells and increased the expression of pro-apoptotic and pro-autophagic proteins in a concentration-dependent manner. The EMT process was reversed after treatment, and the ERK/FOXO3a-dependant mechanism was implicated in these anticancer effects [381]. Luteolin reduced cell viability by inducing apoptosis and this effect was increased in p53-expressing cells. Treatment also reduced oxaliplatin-treated p53-null cell viability and colony counts further and may provide a new treatment for colon cancer cells resistant to oxaliplatin [386]. Luteolin increased the expression of pro-apoptotic proteins and antioxidant enzymes in colon cancer cells [433]. Treatment increased the number of sub-G1 phase cells and cells with fragmented nuclei and increased the expression of the Nrf2 promoter and altered its interaction with the tumor suppressor p53 [433]. Interestingly, one study found that luteolin did not impact cell proliferation of colorectal cancer in vitro or in vivo; however, it inhibited cell migration and invasion. The downregulation of pleiotrophin (PTN) via miR-384 was implicated in luteolin’s effects [390]. In liver cancer cells, luteolin reduced NF-κB transcription factor activation and decreased COX-2 gene expression. It also promoted cytotoxic effects including inhibition of proliferation, ER stress, and induction of apoptosis in a model lacking p53 [382].

The serine-threonine kinase CK2–overexpressed in all cancers where it promotes proliferation, spread, and survival–is inhibited by luteolin [383]. Luteolin induced apoptosis and inhibited the progression of rat prostate carcinogenesis in a transgenic rat for adenocarcinoma of prostate (TRAP) model in addition to a xenograft prostate cancer model where angiogenesis was also inhibited. In another human and rat cell model of prostate cancer, luteolin induced apoptosis by activation of caspases-3 and 7 [387]. Additionally, migration and tumorigenesis were inhibited, and apoptosis was induced in a glioblastoma model. Apoptosis was caused by depolarization of the mitochondrial membrane, ERK protein phosphorylation, cleavage of PARP and caspase-9, causing DNA damage by H2AX phosphorylation [388]. Bladder cancer cell survival was inhibited by luteolin by inducing G2/M cell cycle arrest, p21 upregulation and inhibition of mTOR signalling. In xenograft models, tumor volumes and dimensions were decreased after oral administration of luteolin [377]. When combined with the anticancer agent oxaliplatin, luteolin inhibited gastric cancer cell proliferation and induced apoptosis through the cytochrome-c and caspase pathways and by altering the cell cycle [391].

### 4.6. Orientin

Orientin is a flavone and glucoside derivative of luteolin. Orientin is found in various plants and flowers including the passion flower, bamboo leaves, açaí palm, buckwheat sprouts and in millets. Orientin had anti-migratory and anti-invasive effects in TPA-treated MCF-7 cells. Orientin downregulated TPA-induced membrane translocation of protein kinase C-α, phosphorylation of extracellular signal regulated kinase, and nuclear translocations of activator protein-1 and signal transducer and activator of transcription 3 and inhibited matrix metalloproteinase-9 and IL-8 expression. This resulted in reduced migration and invasion of the MCF-7 cells [392]. Similar inhibitory effects were shown in EC-109 esophageal squamous carcinoma cells in a time and concentration-dependent manner. Orientin-induced apoptosis occurred as a result of upregulated p53 and downregulated pro-apoptotic protein Bcl-2 [393]. Orientin also had cytotoxic and antiproliferative effects against human colorectal cancer HT29 cells. In these cells, orientin induced apoptosis and cell cycle arrest at the G0/G1 phase through the regulation of cyclin and cyclin-dependent protein kinases [394]. Additionally, orientin inhibited T24 human transitional cell bladder carcinoma cell proliferation, induced cell cycle arrest, and decreased the expression of inflammatory mediators such as NF-ĸB and components of the Hedgehog signaling pathway, at a concentration of 100 µM [395]. In vivo, orientin administration (10 mg/kg) resulted in antiproliferative effects on 1,2-dimethyl hydrazine (DMH)-induced colorectal cancer in rats, and improved tumor marker levels while decreasing proliferative marker levels, such as proliferating cell nuclear antigen (PCNA) and Ki67 [396]. Orientin also reduced the occurrence of colonic polyps and aberrant crypt foci and increased the antioxidant defense in rats with DMH-induced colorectal cancer, demonstrating its antiproliferative and antioxidant properties in vivo [397].

### 4.7. Vitexin and Isovitexin

Vitexin and Isovitexin are apigenin flavone glucoside, a chemical compound formed by the combination of apigenin with sugars found in the passion flower, chasteberry, and some bamboo leaves, among other plants. Isovitexin is also known as homovitexin or saponaretin. Anticancer effects have been observed for both these compounds. A review by Ganesan and Xu [434] provides insight into vitexin and isovitexin’s anticancer effects in various in vitro and in vivo models of cancer. The review highlights vitexin and isovitexin’s ability to inhibit cell growth, induce apoptosis, reduce autophagy, reduce cell migration, and provides further mechanistic details about the specific pathways involved. More recently, vitexin has been shown to reduce the viability of adenocarcinomic human alveolar basal epithelial cells dose-dependently, with virtually no toxicity to normal human bronchial epithelial cells. Vitexin induced apoptosis in these cells by increasing pro-apoptotic protein expression, reducing mitochondrial membrane potential and through Akt signalling [398]. In a melanoma model, vitexin inhibited growth in vitro and in vivo, arrested the cell cycle in G2/M phase, induced apoptosis, caused DNA damage, and increased ROS accumulation. Vitexin increased the ROS levels, causing DNA cytotoxicity leading to G2/M cell cycle arrest and apoptosis in BRAFi (BRAF inhibitor)-resistant melanoma cells [399]. Vitexin has shown anticancer effects in several colon cancer models. Chen et al. [402] showed that vitexin promoted apoptosis through p53, p53 upregulated modulator of apoptosis (PUMA) and Bax (Bcl-2-associated X protein) activation. When vitexin was combined with the anticancer agent 5-fluorouracil, a synergistic antitumor effect via PUMA induction was observed. In a multidrug resistant human, colon cancer cell line vitexin had cytotoxic effects by inhibiting autophagy and inducing apoptosis through decreasing autophagy related protein expression and increasing pro-apoptotic protein expression. Additionally, it induced apoptosis and suppressed tumor growth in a multidrug resistant human colon cancer xenograft model [403]. It was also noted that heat shock factor 1 (HSF-1) is a potential target of vitexin in this multidrug resistant model [435]. Both vitexin and isovitexin exhibited anticancer effects in a liver cancer model. These effects were achieved via blocking the STAT3 signaling cascade and reducing survival and invasion. When combined with doxorubicin and sorafenib, vitexin had apoptotic effects [405]. Isovitexin had antiproliferative effects in prostate cancer cells and osteosarcoma cells [400,401]. In the osteosarcoma model, isovitexin further induced apoptosis and caused epigenetic regulation through the DNA methyltransferase 1 (DNMT1)/miR-34a/Bcl-2 axis, causing the suppression of stemness and inducing apoptosis in the spheres derived from osteosarcoma cells. Isovitexin also decreased tumor growth and size in a murine xenograft model [401]. In liver cancer, isovitexin decreased sphere and colony formation rates and stemness-associated markers by downregulating FoxM1 via inhibition of MnSOD. Additionally, isovitexin inhibited liver tumor growth in a murine model [406]. In another hepatocarcinoma model, isovitexin caused miR-34a upregulation, which induced apoptosis and suppressed the stemness of SK-Hep-1 spheroids [404].

### 4.8. Quercetin

Quercetin, a flavonol, is commonly present in red onions, kale, grapes, berries, cherries, broccoli, citrus fruits, as well as in a variety of leaves, seeds, and grains. Quercetin is one of the most abundantly consumed dietary flavonoids, and has a bitter flavor [436]. A recent review by Tang et al. [437] has outlined in detail the anti-cancer actions of quercetin on many types of cancer including, breast, prostate, leukemia, ovarian, gastric, osteosarcoma, melanoma, glioma, lung, colon, and liver in vitro and in vivo. The review highlights the ability of quercetin to inhibit the cell cycle, induce apoptosis through the intrinsic and extrinsic pathways, and inhibit angiogenesis and metastasis in vitro. Quercetin was shown to have the ability to decrease tumor volume and increase survival rate of tumor bearing animals in vivo through apoptosis, and inhibit proliferation, angiogenesis and metastasis [437]. To complement this recent review, we will focus on the anticancer effects of quercetin demonstrated after its publication.

Quercetin treatment reduced cell viability and induced apoptosis in primary and metastatic colon adenocarcinoma cell lines [407]. Quercetin dose-dependently suppressed HGF- and TGFα-induced migration of hepatocellular carcinoma cells by inhibiting the signaling pathway of AKT, but not p38 MAPK [413]. In non-small cell lung carcinoma models, quercetin inhibited proliferation and anchorage-independent growth by inhibiting the Src-mediated Fn14/NF-ĸB pathway both in vitro and in vivo [408] and improved the radiosensitivity of these cells by altering the expression of miR-16-5p and Wee1 [414]. In vivo, lung adenocarcinoma appearance was delayed and increased non-neoplastic body weight gain in mice with tumor oxidative stress after daily treatment with quercetin was observed [420]. In a melanoma model, quercetin treatment decreased proliferation and promoted apoptosis in vivo and in vitro and was found to upregulate IFNα and IFNβ expression through activation of the retinoic acid-inducible gene I promoter [409]. In human prostate cancer cells, in vitro exposure to quercetin downregulated the expression of Metastasis Associated Lung Adenocarcinoma Transcript 1 (MALAT1), suppressed epithelial to mesenchymal transition, promoted apoptosis and inactivated the PI3K/Akt signaling pathway in both a time and dose dependent manner. Similarly, quercetin inhibited the proliferation of prostate cancer in a xenograft model [411,412].

Quercetin in combination with various compounds has been shown to produce enhanced cytotoxic effects in various cancer cells. When combined with the polyphenol resveratrol, quercetin inhibited cell growth and DNA damage, induced S phase cell cycle arrest and caused cell death in oral and pharyngeal cancer cells in a synergistic manner [410]. The combination of curcumin and quercetin lead to synergistic alterations in the expression of genes related to proliferation, apoptosis, cell cycle, inflammation, hypoxia and oxidative stress in myeloid leukemia cancer cells [438] and increased anticancer effects including apoptosis and reactive oxygen species production in breast cancer cells when loaded into apoferritin nanoparticles compared to either free compound alone [439]. Quercetin in combination with *Lycopodium clavatum* extract resulted in reduced cell growth in colon cancer cells. The combined treatment impacted mRNA expression of pro-apoptotic proteins [440].

Quercetin has been shown to have anticancer effects in cell lines resistant to traditional chemotherapeutic agents and in some cases improved the cytotoxicity of those agents. When combined with paclitaxel in a prostate cancer model, quercetin reduced cell proliferation and migration, while apoptosis, G2/M cell cycle arrest, ER stress and ROS were increased. Beneficial effects in a prostate cancer murine model were also observed, where co-treatment of quercetin with paclitaxel increased the effects of paclitaxel, while causing nearly no additional side effects [416]. The combination of doxorubicin and quercetin also produced favorable effects. In colon adenocarcinoma cells overexpressing the P-gp, quercetin improved the cytotoxicity of doxorubicin by inhibiting the ATP-driven transport activity of P-gp and increasing the intracellular accumulation of doxorubicin. It also downregulated the expression of the glutamine transporter solute carrier family 1, member 5 (SLC1A5) [417]. In multi-drug resistant breast cancer cells, the combination of doxorubicin with quercetin reduced cell viability and was mediated through doxorubicin-induced apoptosis. This was shown to decrease in vivo xenograft tumors without producing toxic effects [418]. Similarly, when combined with docetaxel, quercetin caused a reversal of docetaxel resistance in prostate cancer cells through decreased activation of the androgen receptor and PI3K/Akt pathway, fewer mesenchymal and stem-like cell phenotypes and lower P-gp expression. Features altered after treatment included decreased proliferation, colony formation, migration, invasion, and apoptosis. In vivo, xenograft tumors treated with the combination of quercetin and docetaxel had decreased growth [411]. Treatment with quercetin also decreased lymphoid enhancer-binding factor-1 (Lef1) in docetaxel-resistant breast cancer cells and re-sensitized these cells to docetaxel, acting synergistically to reduce the viability of the drug-resistant cells [415]. Quercetin acted in vitro in HeLa cells and in vivo as an inhibitor of the breast cancer resistance protein (BCRP) [421] while it induced cell death in HL60 cells and multidrug resistant HL60/VINC cells [441]. The cell death was mediated through cell cycle arrest, production of reactive oxygen species, caspase mediated apoptosis, and lysosome membrane permeabilization-dependent mechanisms. Finally, Quercetin exhibited cytotoxic and pro-apoptotic effects on gemcitabine-resistant hepatocellular carcinoma and pancreatic cancer cells. The combined treatment of quercetin and gemcitabine lead to an increased pro-apoptotic response, particularly through S phase cell cycle arrest, the upregulation of tumor protein p53 and downregulation of cyclin D1 [419].

## 5. Entourage Effect

It was first suggested by Drs. Mechoulam and Ben-Shabat that the endocannabinoid system demonstrated an effect known as the entourage effect, where a multitude of metabolites and related molecules modified the activity of the endogenous cannabinoids anandamide and 2-arachidonoylglycerol [442]. This proposed concept was further extended to explain how whole botanical drugs were often more effective than their isolated components alone [443,444,445,446], but not everyone is convinced of the existence of this effect. The critics describe this effect as a claim with ill-defined and unsubstantiated pharmacological activities that is “used toward the popularization and sale of ostensible therapeutic products” [447]. It is quite possible that both points of view hold some portion of the truth; where one group focuses on the potential of the multiplicity of compounds resulting in synergistic effects from the compounds present in cannabis despite the effects being mechanistically not well characterized, while the other group observes the lack of extensive research but notices over-interpretations of the results and claims that provide high hopes therapeutically. These claims are supported by recent studies that do not show any effect at the cannabinoid receptors by terpenes. In one study, it was shown that none of the five terpenes tested (myrcene, α- and β- pinene, β-caryophyllene, and limonene; either alone or in mixtures) had direct interactions with CB1 or CB2 receptors [448]. Similarly, another study showed that α-pinene, β-pinene, β-caryophyllene, linalool, limonene, and β-myrcene (up to 30–100 μM) could not directly activate CB1 or CB2, or modulate the signaling of the phytocannabinoid agonist Δ^9^-THC. This suggests that if a phytocannabinoid-terpenoid entourage effect exists, it is not via actions at the CB1 or CB2 receptor level. However, since the reading used involved potassium channels in this study, it remains possible that some of the terpenoids tested could act through other CB1 or CB2-dependent signaling pathways that do not involve potassium channels or simply act through other molecular targets [449]. The hypothesis regarding other targets seems quite plausible; in models of breast cancer, a comparison of pure THC and a botanical extract showed that the botanical drug preparation was more potent than pure THC in producing antitumor responses. The authors reported that the increased potency was not due to the presence of the 5 most abundant terpenes in the preparation (β-caryophyllene, α-humulene, nerolidol, linalool and β-pinene) and that the effects of the botanical preparation modulated different targets and used different mechanisms of action [38].

## 6. Conclusions

Studies of individual pure compounds found in cannabis have demonstrated, as highlighted in this review article, that many of the compounds present in cannabis could be part of a therapeutic solution for specific problems found during cancer treatment. Cannabis has a number of potential benefits especially in the management of symptoms for patients living with and beyond cancer. Cannabis is useful for various symptoms present during cancer like chemotherapy-induced nausea and vomiting, pain and insomnia, for example. While cannabis may be less potent than some other antiemetics, it is sometimes the only drug that works, and it is the only antiemetic that also increases appetite. The potential of being able to use a single preparation that could hold benefit in the treatment of several adverse effects instead of multiple prescriptions that might interact with each other or with cancer-directed therapies seems advantageous.

It is unlikely that consumption of cannabis is sufficient to act as a stand-alone therapy, but rather, formulations that include compounds present in cannabis could very likely generate the beneficial therapeutic effects needed. There are currently several issues that limit the efficacy of anticancer therapies, such as chemotherapeutic resistance, localization of the tumour in hard to reach tissues, or the severe adverse effects of current therapeutic agents. In each of these cases, cannabis could potentially yield effects that could contribute to the efficacy of a therapy or reduce a side effect profile. Examples of this are found in studies that looked at the effects of several flavonoids and some other compounds that were found to block various effector pumps that are often associated with multidrug resistance. Additionally, some terpenes, like borneol, could facilitate the passage of various formulations past the blood-brain barrier for cancers within the brain tissues.

One of the important considerations when suggesting these compounds as therapeutics lies in their potential effects on the activity of not only known cancer therapeutics, but also other compounds found in cannabis. It has been shown that co-treatment of either THC or CBD with carfilzomib can lower the concentration required to have an effect on the viability and migration of cancer cells [77]. Importantly, when the THC and CBD were used in combination the levels of both compounds required to have an effect on carfilzomib was reduced. This then suggests potential synergistic effects of both the cannabinoids with the therapeutic and with each other. As multiple compounds, whether cannabinoids, terpenes or flavonoids have also been shown to display synergistic effects with current chemotherapeutic agents, this may allow for a reduced dosage of each agent required to produce a therapeutic effect, which has the potential to decrease adverse effects experienced by patients from treatments. As noted by some of the critics of this purported entourage effect, there may also be an exacerbation of negative effects, but this would clearly be evaluated within a systematic evaluation of the costs/benefits of combinatorial therapies. Some studies have begun evaluating the potential correlations of different compounds being produced together within cannabis cultivars [322,450,451,452]. It may be possible that clusters of compounds are required to act synergistically. These combinations have given plants unique profiles that may make some better therapeutics. By analyzing the specific chemical characteristics of the most effective cultivars, it may be possible to define and refine the chemicals that are responsible for their therapeutic benefits. This may provide a basis for the optimization of the ratios of compounds used as polytherapies. This is still highly speculative, and it may simply be that the wrong combination of compounds in botanical preparations were used, lacking flavonoids, for example.

With most of the studies up to now having been done in cell lines or animal models, a lot of work remains, in particular in regard to the bioavailability of these plant-derived compounds, before we fully understand the potential benefits of the cannabis polypharmacy in a way that could be used for the treatment of cancer in humans. Additional clinical studies are needed to clarify whether some of these compounds (alone or in combination with other anticancer agents) could be useful in anticancer therapies.

## Figures and Tables

**Table 1:** Anti-Cancer Effects of Cannabinoids Present in Cannabis.

Compound	In Vitro Effects	In Vivo Effects	Clinical Trials
Δ^9^-Tetrahydrocannabinol			
Breast Cancer	Inhibited cell growth and proliferation [30,31,32]. Inhibited estradiol-induced proliferation [31,33]. Increased proliferation and tumor growth [34,35]. Activated transcription factor JunD [32]. Induced apoptosis and cell cycle arrest at G2/M phase [36]. Induced fatty acid 2-hydroxylase [37]. Increased production of reactive oxygen species [38]. Inhibited human P-glycoprotein and breast cancer resistance protein [39].	Increased tumor growth and metastasis [35]. Reduced tumor growth [37,38,40,41]. Inhibited tumor angiogenesis [40].	N/A
Brain Cancer	Inhibited cell viability and proliferation dose-dependently [42,43,44,45]. Induced apoptosis [46,47,48,49]. Stimulated glioma cell growth [50]. Induced autophagy via ceramide accumulation and ER stress [47,51,52]. Down-regulated expression of matrix metalloproteinase-2 [53]. THC + CBD pre-exposure increased sensitivity to radiation therapy [53].	Reduced tumor growth [47,48,51,52]. Upregulated stress protein p8 and induced apoptosis [47]. Induced autophagy [48]. THC + Temozolomide synergistically reduced growth of xenograft tumors [54,55,56]. Down-regulated expression of metalloproteinase-2 [53]. THC-loaded nanoparticles reduced cell proliferation, angiogenesis and increased apoptosis [57].	Pilocytic astrocytoma tumors regressed over a period of 3 years following the inhalation of cannabis over the same period [58]. Temozolomide + Sativex increased 1-year survival rate in GBM patients [NCT01812603 and NCT01812616]. Reduced tumor cell Ki67 staining in patients suffering from recurrent GBM [59]. Reduced VEGF and VEGFR-2 activation in GBM patients [60]. Dronabinol treatment did not lead to severe adverse effects in patients with primary brain tumors [61].
Leukemia	Reduced proliferation and exhibited cytotoxicity [62]. Sensitized leukemia cells to anti-cancer agents [62,63,64]. Inhibited the differentiation blockage (Dronabinol) [65]. Induced apoptosis [66,67,68]. Induced apoptosis in patient-derived leukemia cells [66]	N/A	Remission achieved following the consumption of *Cannabis sativa* oil in a patient with terminal acute lymphoblastic leukemia [69]. Dronabinol inhibited the differentiation blockage in leukemia patients [66].
Lung Cancer	Low levels induced cell proliferation or did not decrease cell survival [50,70]. Inhibited cell proliferation, chemotaxis and invasion [71,72]. Reduced migration [72]. Inhibited host immune response and killing of tumor cells [73]. Suppressed EMT of NSCLC cells [72]. THC-loaded nanoparticles exhibited cytotoxicity [74].	Increased tumor growth and reduced tumor immunogenicity [75]. Inhibited tumor growth and metastases [71]. THC-loaded nanoparticles exhibited significant cytotoxicity [74].	N/A
Melanoma & Myeloma	Inhibited growth and proliferation [76,77]. Induced apoptosis and autophagy [78]. Induced autophagic-dependent necrosis [77]. THC + CBD had synergistic effects with carfilzomib [77]. Increased cell death and decreased migration [77].	Reduced proliferation, metastasis, angiogenesis, tumor growth and increased apoptosis [76,79]. THC:CBD in a 1:1 ratio decreased tumor growth and increased autophagy and apoptosis [78]. THC + Trametinib reduced viability, invasion and metastasis of MEKi-resistant melanoma cells [80]. Induced myeloid-derived suppressor cell function and differentiation [81].	N/A
Hepatocellular Carcinoma	Decreased cell viability and induced autophagy [82]. Increased activity of PPARγ [83]. Reduced proliferation, migration, invasion, and induced apoptosis [84].	Reduced tumor growth [82]. Increased the activity of PPARγ [83]. THC + Irinotecan reduced hepatic toxicity during acute treatment [85].	N/A
Pancreatic, Prostate, Colon Cancer	Decreased cell viability [30,86,87]. Induced apoptosis [86,88,89]. THC-loaded microspheres inhibited proliferation [90].	Reduced the growth of tumors [86].	N/A
Endometrial, Cervival, Oral Cancer	Increased accumulation of anti-cancer agents in cells expressing multi-drug transporters [91,92]. Reduced invasion via increased TIMP-1 expression [93]. Inhibited mitochondrial oxygen consumption and exhibited strong toxicity [94].	N/A	N/A
Cannabidiol			
Breast Cancer	Induced apoptosis and autophagy [30,95]. Enhanced production of reactive oxygen species and subsequent ER stress [95]. Inhibited proliferation, migration and invasion [96,97,98]. Inhibited the EMT and reduced expression of malignant markers [98]. Increased sensitivity to anti-cancer agents doxorubicin and cisplatin [98]. Synergistic effects with paclitaxel and doxorubicin on antiproliferative activity [99].	Inhibited tumor growth, migration, invasion, and metastasis [97]. Increased survival and decreased metastasis [100]. Down-regulated Id1 expression [100].	N/A
Lung Cancer	Induced apoptosis [101]. Reduced invasion, metastasis, migration, and restored epithelial phenotype [72,102,103,104]. Increased susceptibility to lysis by lymphokine-activated killer cells [105].	Reduced cell viability [101]. Decreased tumor growth [101,103]. Decreased metastasis [103].	N/A
Glioma & Neuroblastoma	Inhibited cell proliferation and induced apoptosis [43,106,107,108,109,110,111]. Increased reactive oxygen species production [110,112]. Increased expression of heat shock proteins [112]. Induced cell cycle arrest [111]. Reduced invasion [109,111].	CBD + THC + Temozolomide reduced tumor growth [54,55]. Reduced tumor growth [57,108]. Enhanced apoptosis and decreased angiogenesis [57]. Significantly prolonged mouse survival [110].	Sativex + Temozolomide increased the rate of 1-year survival by 39 percent in GBM patients [NCT01812603 and NCT01812616].
Colon & Prostate Cancer	Induced apoptosis, cell cycle arrest and ROS production [87]. Reduced cell proliferation, promoted apoptosis and elevated ROS levels [87,113,114,115,116]. Antagonistic activity at GPR55 reduced and prevented metastasis [117].	Increased effects of anti-cancer agents bicalutamide and docetaxel [87]. Reduced aberrant crypt foci polyps and tumor growth [87,113,114,118]. Chemo-preventative on colon cancer cells due to up-regulated caspase-3 [113]. Decreased metastasis and angiogenesis [114].	N/A
Myeloma, Melanoma, Leukemia	Reduced cell viability [77,119]. Induced apoptosis due to ceramide accumulation [120]. Decreased P-glycoprotein expression and sensitized cells to Vinblastine [64]. Increased cytotoxicity of bortezomib and carfilzomib [77,119].	Increased mouse survival and reduced tumor growth [78,116].	N/A
Cervical, Endometrial, Ovarian Cancer	Inhibited cell growth and induced apoptosis [121,122]. Increased intracellular accumulation of multi-drug transporter substrates Fluo3, vincristine, and mitoxantrone [91,92].	N/A	N/A
Cannabigerol	Significant inhibitory effects on cell proliferation [123,124,125]. Inhibited [^14^C]anandamide uptake and activated TRPV1 receptor [30]. Stimulated apoptosis and ROS production [123].	Decreased tumor growth due to antagonistic activity at TRPM8 receptors [123].	N/A
Cannabichromene	Inhibited cell viability and growth [30,87,123]. Significantly activated caspase 3/7 [87]. Elevated intracellular Ca^2+^ levels [87].	N/A	N/A
Cannabidivarin	Dose-dependent inhibitory effects on cell viability [87,123].	N/A	N/A
Cannabinol	Cytotoxic effects at high concentrations [87]. Antiproliferative effects [96]. Inhibited multi-drug transporter ABCG2 and promoted accumulation of mitoxantrone [92].	N/A	N/A
Tetrahydrocannabivarin	Cytotoxic effects at higher concentrations [87].	N/A	N/A

**Table 2:** Anti-Cancer Effects of Terpenes Present in Cannabis.

Compound	In Vitro effects	In Vivo effects	Clinical Trials
**Myrcene**	Exhibited cytotoxic effects [145,146,147,148]. Decreased DNA damage [149].	Carcinogenic [150].	N/A
**β-Caryophyllene and metabolite caryophyllene oxide**	Exhibited cytotoxic effects [151,152]. Induced apoptosis [151,152,153,154]. Induced cell cycle arrest [151,152,154]. Increased ROS production [153]. Activated the JAK1/STAT3 pathway [153,155]. Enhanced doxorubicin sensitivity [155,156,157,158,159]. Enhanced 5-fluoruracil sensitivity [160]. Enhanced oxaliplatin sensitivity [160]. Enhanced sorafenib sensitivity [161].	Decreased doxorubicin-induced cardiotoxicity [162].	N/A
**Humulene**	Exhibited cytotoxic effects [163,164,165]. Increased ROS production [163]. Inhibited Akt activation [164]. Enhanced effects of 5-fluoruracil [160]. Enhanced effects of oxaliplatin [160]. Enhanced effects of doxorubicin [158].	Inhibited cell proliferation [164]. Increased occurrence of apoptosis [164].	N/A
**Limonene**	Exhibited cytotoxic effects [166,167,168]. Induced cell cycle arrest [166]. Decreased migration [166]. Decreased invasion [166]. Induced apoptosis [166,167,169,170,171]. Inhibited the PI3K/Akt pathway [167]. Induced autophagy [172,173,174]. Enhanced sensitivity to docetaxel [171].	Decreased tumor growth [174,175,176,177,178]. Induced apoptosis [179,180,181,182,183]. Increased latency period [175,176,177,183]. Decreased c-jun and c-myc expression [184]. Decreased metastasis [180,181,182,185].	Decreased cell cycle regulatory protein expression in human tumors [186].
**Pinene**	Reduced cell viability [187,188,189,190]. Induced apoptosis [187,188,189,191,192]. Increased ROS production [188,191]. Induced cell cycle arrest [187,188,192,193,194]. Acted synergistically with paclitaxel [192].	Reduced tumor growth [191]. Reduced the number of tumor nodules [191].	N/A
**Linalool**	Reduced cell viability [195,196,197,198,199]. Induced apoptosis [195,197,200,201]. Induced cell cycle arrest [195,196,198,199]. Decreased p-Akt and PI3K expression [195]. Increased expression of pro-apoptotic proteins Bax, Bak, caspase-2, caspase-9 [197]. Decreased expression of Bcl-2 and Bcl-xl [197]. Increased doxorubicin sensitivity by increasing doxorubicin influx [202,203].	Reduced xenograft tumor volume [200,201]. Caused tumor specific lipid peroxidation [200]. Reduced tumor incidence following UVB-exposure [204]. Acted synergistically with doxorubicin to decrease tumor weight in mice [202].	N/A
**Bisabolol**	Exhibited cytotoxic effects and inhibited cell growth [205,206,207,208,209,210,211,212,213,214,215,216]. Induced apoptosis [217]. Induced autophagy [217]. Inhibited the PIK3/Akt signalling pathway [206]. Increased sensitivity to radiotherapy [213].	Not generally toxic in murine models [217]. Decreased number of palpable tumor masses [218]. Inhibited xenograft tumor growth [219]. Increased 5-aminolevulinic acid retention in buccal tissue [220].	N/A
**Elemene**	Induced cell cycle arrest [216,221,222,223,224,225]. Induced apoptosis [216,221,222,223,224,225,226]. Inhibited MAPK and PI3K/Akt/mTOR signalling [226]. Reduced invasion and metastasis [226]. Inhibited angiogenesis [226]. Enhanced sensitivity to several chemotherapeutic agents [227,228,229,230,231]. Increased sensitivity to paclitaxel, colchicine, and vinblastine through ABCB1 inhibition [232,233].	N/A	Injection shown to be effective adjective treatment to platinum-based chemotherapy [234]. Reduced toxicity of chemotherapy [234]. Positive effect in combination with chemotherapy in several cancer types [235].
**Eudesmols**	Exhibited cytotoxic effects [236,237]. Inhibited cell proliferation [238]. Inhibited superoxide production [238]. Inhibited adhesion and migration [238]. Induced apoptosis [239,240]. Induced cell cycle arrest [239,240]. Enhanced cytotoxicity of 5-fluoruracil [241]. Enhanced anti-migratory effects of doxorubicin [241].	Reduced tumor growth [237,242]. Increased survival [242]. Reduced metastasis [242].	N/A
**Eucalyptol**	Exhibited cytotoxic effects [148,243]. Induced apoptosis [244,245,246,247]. Induced cell cycle arrest [245,247]. Changed gene expression of MDM4, NF-_k_B, and VEGF in ANXA7 expressing cells [243]. Inactivated survivin and Akt [246]. Activated p38 [246]. Interacted with Bcl-2 and PARP1 receptor [247].	Reduced tumor progression in xenograft tumors [246].	N/A
**Borneol**	Enhanced doxorubicin induced cell cycle arrest [248]. Increased doxorubicin-induced interference with MAPKs and PI3K/Akt pathways in vitro and in vivo [248]. Enhanced prop-apoptotic effects of paclitaxel [249]. Increased cellular uptake of selenocystine [250]. Acted synergistically with curcumin-based drugs [251,252]. Acted synergistically with temozolomide [253]. Enhanced doxorubicin delivery in vitro and in vivo using borneol modified nanomicelles [254]. Enhanced chemotherapeutic effects when loaded in nanomolecule formulations [255,256,257,258].	Induced transient disruption of the blood-brain barrier [259].	N/A
**Terpineol**	Exhibited cytotoxic effects [260,261]. Inhibited cell proliferation [262,263]. Induced apoptosis [263]. Induced cell cycle arrest [261,263]. Reduced cell migration [261]. Potentiated the effects of oxaliplatin and 5-fluoruracil [264].	Reduced tumor weight and volume [261].	N/A
**Terpinene isomers**	Reduced proliferation [265,266,267,268]. Induced apoptosis [267,268].	N/A	N/A
**Valencene**	Reduced cellular proliferation [158,269]. Acted synergistically with doxorubicin to reduce proliferation [269].	N/A	N/A
**Geraniol**	Reduced cellular proliferation [270,271]. Induced apoptosis [270]. Induced cell cycle arrest [271,272]. Downregulated Blc-2 and upregulated Bax [270,271].	N/A	N/A
**Nerolidol**	Exhibited cytotoxic effects [273,274,275]. Induced apoptosis [159,276]. Induced cell cycle arrest [276]. Decreased adhesion of TNF-α induced cells [159]. Acted synergistically with doxorubicin to reduce cell viability [159,269].	Inhibited azoxymethane induced cancer [277].	N/A
**Guaiol**	Reduced cell proliferation [278,279]. Reduced metastasis [278]. Inhibited mTORC2-Akt signalling to induce autophagy [280].	Reduced cell growth [279].	N/A
**Camphene**	Inhibited cell proliferation [281]. Induced apoptosis [281].	Reduced subcutaneous tumor growth in a syngeneic model [281].	N/A
**α-phellandrene**	Decreased cell viability [282]. Induced cell cycle arrest [194]. Altered expression of genes involved in apoptosis, DNA damage, and cell cycle [283]. Increased expression of phosphorylated p53, phosphorylated-H2A.X, 14-3-3-σ, and MDC1 [284,285]. Decreased expression of p53, MGMT, DNA-PK, and BRCA-1 [284]. Increased ROS production [282,286]. Induced autophagy [285]. Induced necrosis [282,285].	N/A	N/A
**Δ-3-carene**	Cytotoxic when found in essential oil extracts [287,288].	N/A	N/A
**Cadinenes**	Cytotoxic when found in essential oil extracts [289,290,291]. Inhibited cell growth [292]. Induced apoptosis [292]. Induced cell cycle arrest [292].	N/A	N/A
**Thujone**	Exhibited cytotoxic effects [293]. Induced apoptosis [293].	Brain, liver, and kidney toxicity [294].	N/A
**p-Cymene**	Anti-tumor effects when found in essential oils from *Nigella sativa* [295]. Ruthenium^11^(p-Cymene) complexes were effective and selective against several cancers [296,297,298].	N/A	N/A
**Gurjunene**	Inhibited cell growth when found in an essential oil extract [299,300,301]. Induced apoptosis [299]	N/A	N/A
**Farnesene**	Induced cell death when found in an essential oil extract [302,303,304]. Essential oil *Garcinia atroviridis* acted synergistically with tamoxifen [302].	N/A	N/A

**Table 3:** Anti-Cancer Effects of Flavonoids Present in Cannabis.

Compound	In Vitro Effects	In Vivo Effects	Clinical Trials
**Kaempferol**	Inhibited cell viability in a dose-dependent manner [327,328,329,330,331,332,333,334,335]. Induced cell cycle arrest at the G2/M or G0/G1 phase [329,335,336,337,338,339,340]. Reduced migration and invasion [340,341,342,343,344,345,346]. Inhibited the EMT and reduced resistance to chemotherapeutic agents [343,347]. Altered expression of VEGF [348]. Induced apoptosis and autophagy [330,340]. Kaempferol + Luteolin inhibited cell proliferation, induced cell death, inhibited migration and invasion [349]. Kaempferol + TRAIL induced apoptosis [350]. Kaempferol + 5-fluorouracil had synergistic anti-proliferative effects and re-sensitized resistant cells to chemotherapeutic agents [341,351].	Increased mouse survival [339,352,353,354,355]. Reduced tumor growth and metastasis [339,352,353,354,355,356]. Caused degranulation and accumulation of mediators in leukemia cells [357,358].	N/A
**Apigenin**	Reduced cell viability and proliferation [359,360,361]. Induced cell cycle arrest at the G1 or G2/M phase [360,362,363]. Inhibited hypoxia-induced resistance via suppression of HIF-1α [362]. Enhanced activity of paclitaxel [362]. Apigenin + Sorafenib increased apoptosis and decreased migration and invasion [364]. Apigenin + Abivertinib had synergistic anti-cancer effects [360]. Induced apoptosis and reduced angiogenesis [361].	Exacerbated the effects of paclitaxel [362]. Inhibited tumor growth via ER-mediated PI3K/Akt/mTOR pathway [365]. Apigenin + Abivertinib exhibited synergistic anti-cancer effects [360]. Apigenin combined with IL-6 inhibition potentiated anti-cancer effects of apigenin [365].	N/A
**Cannflavin B**	Increased apoptosis [366].	Delayed local and metastatic tumor progression [366]. Increased survival [366].	N/A
**Silymarin**	Induced apoptosis [367,368,369,370]. Reduced cell viability and proliferation [368,370]. Silymarin nanoemulsion + cold atmospheric plasma reduced intracellular ATP levels and down-regulate transcriptional and survival pathways [371]. Inhibited EMT and migration [372].	Reduced tumor volume and induced apoptosis [369].	High dose silibinin was well tolerated in patients; common adverse event observed was asymptomatic liver toxicity [373].
**Luteolin**	Caused cell cycle arrest [362,374,375,376,377]. Decreased cell viability and proliferation [378,379,380,381,382,383]. Inhibited the EMT [376,381,384]. Inflicted double-stranded DNA breaks and prevented nonhomologous end joining [385]. Induced apoptosis [374,375,382,386,387,388]. Reduced migration and invasion [378,379,388,389,390]. Luteolin + Oxaliplatin inhibited proliferation, induced apoptosis and altered the cell cycle [391].	Inhibited cell growth [378]. Reduced migration, invasion and metastasis [384]. Inhibited angiogenesis [387]. Decreased tumor volume and dimension [377].	N/A
**Orientin**	Reduced migration and invasion [392]. Induced apoptosis and altered apoptotic protein levels [393,394]. Caused cell cycle arrest [394,395]. Decreased cell proliferation [393,395].	Antiproliferative effects [396]. Improved tumor marker levels and decreased proliferative marker levels [396]. Reduced occurrence of polyps and aberrant crypt foci [397]. Increased antioxidant defense [397].	N/A
**Vitexin & Isovitexin**	Reduced cell viability and proliferation [398,399,400,401]. Induced apoptosis [398,399,401,402,403,404]. Caused cell cycle arrest at the G2/M phase [399]. Vitexin + 5-fluorouracil had synergistic anti-tumor effects via PUMA induction [402]. Vitexin + Doxorubicin + Sorafenib induced apoptosis [405].	Inhibited cell/tumor growth [399,401,403,406]. Induced apoptosis [403]. Reduced overall tumor size [401].	N/A
**Quercetin**	Decreased cell viability and proliferation [407,408,409,410]. Induced apoptosis [407,409,411,412]. Reduced migration [413]. Increased the radiosensitivity of cells [414]. Reversed docetaxel resistance [411,415]. Inhibited the EMT and downregulated expression of MALAT1 [412]. Quercetin + Paclitaxel reduced cell proliferation, migration, and induced apoptosis and cell cycle arrest [416]. Quercetin + Doxorubicin caused increased cytotoxicity and induced apoptosis [417,418]. Quercetin + Gemcitabine caused increased apoptosis in gemcitabine-resistant cancer cells [419].	Inhibited cell proliferation and tumor growth [408,411,412]. Delayed appearance of lung adenocarcinoma [420]. Reversed docetaxel resistance [411]. Inhibited breast cancer resistance protein [421]. Quercetin + Paclitaxel increased anti-cancer effects of paclitaxel [416]. Quercetin + Doxorubicin decreased tumor growth [418]. Quercetin + Docetaxel decreased tumor growth [411].	N/A
